# Dynamic changes in gut microbiota of three breeds of stud boars pre- and post-amoxicillin administration

**DOI:** 10.3389/fvets.2026.1930671

**Published:** 2026-10-01

**Authors:** Liusheng Wang, Huiting He, Tao Huang, Zhelong Jiang

**Affiliations:** 1 Department of Respiratory and Critical Medicine, Affiliated Hangzhou First People’s Hospital, School of Medicine, Westlake University, Hangzhou, China; 2 Key Laboratory of Applied Technology on Green-Eco-Healthy Animal Husbandry of Zhejiang Province, College of Animal Science and Technology, College of Veterinary Medicine, Zhejiang A&F University, Hangzhou, China; 3 Department of Emergency, Affiliated Hangzhou First People’s Hospital, School of Medicine, Westlake University, Hangzhou, China

**Keywords:** 16S rRNA, amoxicillin, boars, dynamic trajectory, fecal microbiota

## Abstract

**Introduction:**

Amoxicillin is a commonly used broad-spectrum antibiotic in pig farming, valued for its cost-effectiveness and efficacy in addressing prevalent swine ailments. However, the specific effects on the gut microbiota of stud boars and the trajectory of microbial recovery post-antibiotic withdrawal remain insufficiently elucidated.

**Method:**

This study aimed to investigate the dynamic trajectory of gut microbiota in 40 Large White (LW), 47 Landrace (LAN), and 16 Duroc (DUR) stud boars before (D0), during (D7), and after (D14) amoxicillin administration (100 mg/kg of feed from D3 to D7) using 16S rRNA sequencing.

**Results:**

Amoxicillin significantly reduced alpha diversity (measured by observed species and the Shannon index) in LW and LAN stud boars at D7, with partial recovery by D14. In contrast, DUR stud boars showed no significant changes in diversity. Principal coordinate analysis revealed distinct microbial community structures across different time points, with samples from D7 forming a separate cluster, distinct from those collected on D0 and D14. At the genus level, *Lactobacillus* and *Streptococcus* were predominant in LW and LAN, with relative abundances increasing during amoxicillin treatment and decreasing post-withdrawal. In DUR stud boars, *Prevotella* and *Lactobacillus* were more abundant. Functional predictions indicated changes in KEGG pathways related to metabolism and drug resistance. These findings highlight the breed-specific responses of gut microbiota to amoxicillin. While LW and LAN showed signs of recovery after the withdrawal of the antibiotic, DUR exhibited resilience.

**Conclusion:**

Taken together, this study aids in optimizing antibiotic use in pig farming, reducing resistance risks, and promoting sustainable animal health.

## 1 Introduction

Amoxicillin is a 𝛽-lactam antibiotic that targets bacterial enzymes involved in cell wall formation. It is the most frequently prescribed broad-spectrum antibiotic globally (1). It can exert bactericidal effects by inhibiting bacterial cell wall synthesis, which can quickly cause bacteria to become spherical, dissolve, or rupture (2). Amoxicillin is considered an extremely valuable antimicrobial in human and animal medicine. Discovered in 1970, amoxicillin remains the most widely used antibiotic in the pig industry due to its cost-effectiveness and good oral absorption (3). In pig farming, amoxicillin is effective in treating diseases such as swine erysipelas, bacterial respiratory infections, enteritis, and diarrhea (4, 5). Therefore, research on the effects of amoxicillin on pigs is crucial for the advancement of the pig farming industry.

Amoxicillin treatments have drawbacks that can negatively impact beneficial resident microbes in the human body (6, 7). Oral amoxicillin can significantly disrupt the gastrointestinal microbiota, leading to widespread changes in species-level composition, a condition known as dysbiosis (8). This disruption can lead to a decrease in colonization resistance against various fungal and bacterial pathogens, increasing the risk of opportunistic infections (9, 10). In addition to the increased risk from pathogens, dysbiosis can have a substantial negative effect on the critical role of the gastrointestinal microbiota in host immunity, metabolism, and even neurological function (11, 12). Long-term amoxicillin treatment may exert selective pressure on the gut microbiota, increasing the diversity of antimicrobial resistance genes and promoting the establishment of a more diverse resistant population (13, 14). Early exposure to amoxicillin in pigs can result in systemic expression of pro-inflammatory genes, while amoxicillin administration to mother pigs can affect the metabolic products and colon microbiota of piglets through their milk (15, 16).

Treatment with amoxicillin has been shown to induce rapid reductions in bacterial counts and diversity within the microbiome (17). Specifically, it can result in a loss of anaerobic *Firmicutes* and/or *Bacteroidetes*, with a concomitant expansion of *Proteobacteria* and other facultative anaerobes (6). In healthy adults who take amoxicillin, the abundance of *Clostridiales*, *Lachnospiraceae*, and *Coriobacteriaceae* decreases, while *Porphyromonadaceae*, *Bacteroidaceae*, and *Enterobacteriaceae* increase (18). In addition, dogs treated with amoxicillin have an increased incidence and proportion of ampicillin-resistant enterococci, with a significant increase in the total count of enterococci compared to their levels before treatment (19). Furthermore, in a study by Locke and Larsen on Brown Norway rats, the abundance of *Proteobacteria* and *Bacteroidetes* increased, while the abundance of *Firmicutes* decreased sharply after amoxicillin administration (20). Similarly, treatment of piglets with amoxicillin leads to a higher abundance of *Proteobacteria* and a decrease in *Firmicutes* (15). Taken together, amoxicillin can disrupt the gut microbiota by altering its community composition and structure, typically leading to a significant reduction in alpha diversity.

To date, many studies have elucidated the effects of amoxicillin treatment on the gut microbiota (8, 21). Many of these studies have focused on the effects observed in mice and humans. However, there is limited research on the short-term effects of amoxicillin on the intestinal microbiota of pigs and the recovery process after treatment discontinuation (5, 22, 23). Furthermore, these investigations lack early sampling time points before medication administration and have very extensive experimental durations with late sampling times. This could have resulted in the oversight of important details that might enhance authors’ insights. In our study, we have, for the first time, revealed the dynamic trajectory in the composition and structure of gut microbiota before and after amoxicillin administration in three breeds of boars. Therefore, we reared three different breeds of stud boars under the same conditions, administered amoxicillin on day 3, and discontinued it on day 7. We collected fecal samples on days 0, 7, and 14 for analysis. This study aims to investigate the effects of short-term administration of amoxicillin on intestinal flora and its recovery in pigs, which is valuable for understanding the application of amoxicillin in the pig industry and for promoting disease prevention and treatment strategies in these animals.

## 2 Materials and methods

### 2.1 Animal experimental design and sample collection

A total of 103 stud boars from 3 different breeds were used as experimental animals in this study. The breeds included 40 Large White (LW), 47 Landrace (LAN), and 16 Duroc (DUR) boars. All were reared at a specialized breeding pig testing center located in Lin’an, Zhejiang, China. These stud boars were 50–55 days old at the start of the experiment (D0). To ensure the accuracy of the experiment, all stud boars were housed in the same building and managed under identical feeding and care conditions. The pen size was 3 m × 2.5 m, with approximately 10 stud boars of the same breed housed per pen. The boars were fed twice a day at 8:00 a.m. and 4:00 p.m., with ad libitum access to feed and water. The growth, development, nutritional status, appetite, and body condition of all boars involved in this experiment were normal. No antibiotics were used before the start of this experiment.

All stud boars were healthy prior to the start of the experiment, which was designated as Day 0 (D0). Amoxicillin was introduced into their diet at a dose of 100 mg/kg of feed, starting on day 3 (D3), with all other conditions remaining unchanged. The administration of amoxicillin was stopped on the seventh day (D7) of the experiment; the standard basal diet was used until the 14th day (D14). Fresh fecal samples were collected on days 0, 7, and 14. A sterile cotton swab was inserted approximately 1 cm into the anus to induce defecation. Freshly excreted feces were directly collected using sterile gloves to prevent contamination from the floor. Approximately 1 cm^3^ of each fecal aliquot was immediately transferred to a 1.5 mL cryovial, snap-frozen in liquid nitrogen, and then stored at −80 °C until DNA extraction (Figure 1A).

**Figure 1:**
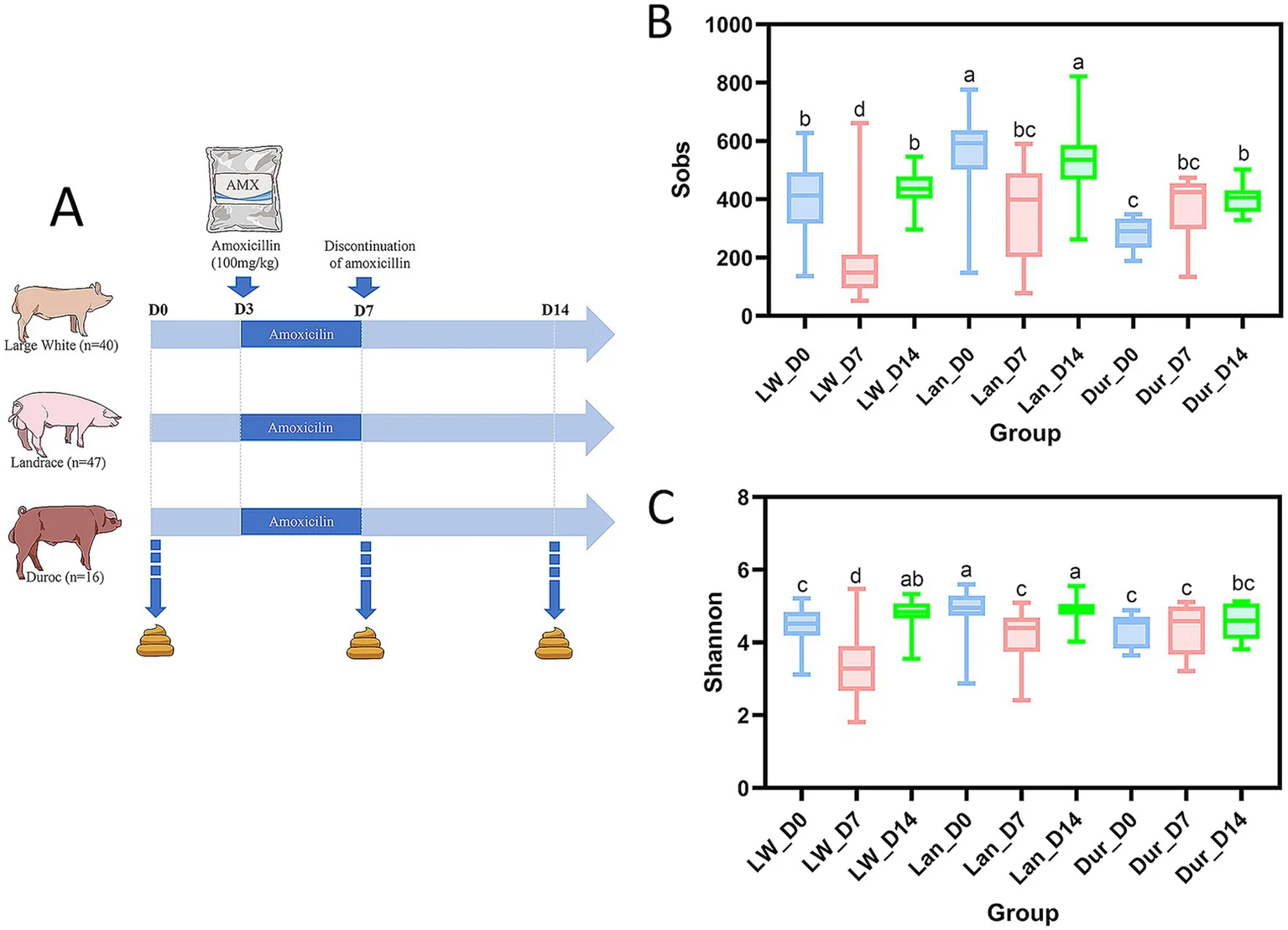
Experimental design and alpha diversity of gut microbiota. **(A)** Experimental designs for three boar breeds before, during, and after amoxicillin administration. **(B)** Sob index. **(C)** Shannon index. LW, Large White; LAN, Landrace; DUR, Duroc. Different lowercase letters above boxplots indicate statistically significant differences between groups (*p* < 0.05). Groups sharing the same letter are not significantly different from one another; groups with different letters are significantly different.

### 2.2 DNA extraction and 16S rRNA gene amplicon sequencing

DNA was extracted from fecal samples using the QIAamp DNA Stool Mini Kit (Qiagen, Hilden, Germany) following the manufacturer’s protocol. Genomic DNA concentration and quality were assessed with a NanoDrop 2000 spectrophotometer (Thermo Fisher Scientific, Waltham, MA, USA). The V3-V4 hypervariable region of the bacterial 16S rRNA gene was amplified with universal primers 338F (5′-ACTCCTACGGGAGGCAGCAG-3′) and 806R (5′-GGACTACHVGGGTWTCTAAT-3′). Polymerase chain reaction (PCR) was performed on an ABI 9700 instrument (Thermo Fisher Scientific, Waltham, MA, USA) in a 25 μL reaction mixture containing 3 μL bovine serum albumin (BSA) (2 ng/μL), 1 μL of each primer (5 μM), 2 μL template DNA, 12.5 μL 2 × Taq Plus master mix, and 5.5 μL ddH_2_O. Cycling conditions included 95 °C for 5 min, 28 cycles of 95 °C (45 s), 55 °C (50 s), and 72 °C (45 s), and a final extension at 72 °C for 10 min. PCR products were purified using the Agencourt AMPure XP Kit (Beckman Coulter, Inc., CA, USA). Libraries were constructed with the NEB Next Ultra II DNA Library Preparation Kit (New England Biolabs, Inc., MA, USA) and sequenced on the Illumina MiSeq platform (Illumina, CA, USA).

### 2.3 16S rRNA sequence data analysis

Raw data were filtered and spliced using Trimmomatic, and FLASH v1.2.11 (Magoc and Salzberg, Johns Hopkins University, USA) was used for merging (24). The criteria were as follows: sequences containing ambiguous bases (N), quality scores < 20, or lengths < 230 bp were excluded. Chimeras were removed via UCHIME against the Gold Database. Sequences were clustered into amplified sequence variants (ASVs) using DADA2 to obtain representative sequences and abundance data, annotated against the Silva 138 Database (24–26). The alpha-diversity index and beta-diversity based on Bray-Curtis distance were analyzed in QIIME2 (QIIME 2 Development Team, University of California, San Diego, La Jolla, CA, USA) (27). Principal coordinate analysis (PCoA) of Bray-Curtis distances and permutational multivariate analysis of variance (PERMANOVA) assessed sample clustering using the vegan package (v.2.6–4) in R (28, 29). Microbiota functional profiles were inferred using PICRUSt2 (v2.2.0; Langille Lab, McGill University, Montréal, Québec, Canada) (30).

### 2.4 Statistical analysis

All statistical tests were performed in R (v 4.3.1) (29) unless otherwise stated. The Wilcoxon rank-sum test with false discovery rate (FDR) correction was used for pairwise comparisons between the groups, and the Kruskal–Wallis test was employed for comparisons between multiple groups.

## 3 Results

### 3.1 16S rRNA sequencing data of all fecal samples

In this work, we generated a novel resource of the dynamic trajectory of gut microbiota in three breeds of stud boars before, during, and after amoxicillin feeding, comprising a total of 279 fecal samples from 40 Large White, 47 Landrace, and 16 Duroc stud boars (Figure 1A). Amplicon sequencing based on the 16S rRNA V3-V4 hypervariable region yielded 17,045,354 raw sequences. The average sequence number for LW, LAN, and DUR samples was 58985.14 ± 11076.38, 61737.79 ± 10268.40, and 57292.45 ± 9469.79, respectively. Based on these sequences, a total of 8,110 ASVs were detected. The ASVs were classified according to microbial taxonomy, and 19 phyla and 327 genera were identified (Supplementary Figure S1; Supplementary Table S1).

### 3.2 Microbial diversity at different time points in three breeds of stud boars fed amoxicillin

To compare the alpha-diversity of gut microbiota at different time points of amoxicillin feeding, we calculated the observed species index and the Shannon index at the ASV level in three breeds of stud boars (Figures 1B,C). In LW stud boars, the observed species and the Shannon indices during amoxicillin feeding (D7) were significantly different from before (D0) and after amoxicillin feeding (D14) (*p <* 0.001). The observed species and Shannon indices of D7 were found to be significantly lower than those of D0 and D14 (*p <* 0.001), but the observed species indices were not significantly different between D0 and D14 in LW stud boars (*p* = 0.24). In LAN stud boars, we observed the same results as in the LW stud boars, with significant differences in the observed species and Shannon indices at D7 compared to D0 and D14, respectively (*p <* 0.001). The observed species and Shannon indices were the lowest at D7 compared with D0 and D14 in the LAN stud boars. Between D0 and D14 of LAN stud boars, there were no significant differences in observed species (*p* = 0.16) and Shannon indices (*p* = 0.29). The DUR boars showed no significant differences in either the observed or Shannon indices across the sampling time points.

To investigate the differences in the effects of amoxicillin feeding on the gut microbiota of stud boars, we compared the alpha-diversity of gut microbiota among three breeds of stud boars administered amoxicillin at each time point. Before amoxicillin feeding (D0), the observed species and the Shannon indices were significantly different among LW, LAN, and DUR breeds (*p <* 0.001), among which there were significant differences in observed species and Shannon indices between pairs of breeds (*p <* 0.05), except for the lack of a significant difference in Shannon index between LW and DUR breeds (*p* = 0.68) (Figures 1B,C). The LAN stud boars had the highest observed species and Shannon indices before amoxicillin feeding. During amoxicillin feeding (D7), there were significant differences in observed species and Shannon indices among the three breeds of stud boars (*p <* 0.001), and we found no significant differences between LAN and DUR. The observed species and Shannon indices of D7 in DUR were the highest, whereas those in LW were the lowest. After amoxicillin feeding (D14), there were significant differences in the Sobs index among the three breeds of stud boars (*p* = 2.93 × 10^−7^), but no significant differences in the Shannon index (*p* = 0.06). The LAN stud boars had the highest observed species and Shannon indices after amoxicillin feeding.

### 3.3 Structure of the microbial community in stud boars fed amoxicillin

Principal coordinate analysis (PCoA) at the ASV level was used to compare the degree of similarity in the overall structure of the microbial community (Figure 2). Samples clustered significantly according to breed and time points of amoxicillin feeding (PERMANOVA, *p <* 0.001) (Figure 2A). For LW stud boars, the samples exhibited significant clustering according to the times of amoxicillin feeding (PERMANOVA, *p <* 0.001) (Figure 2B). The D7 samples were clustered separately from the other groups, whereas the D0 and D14 samples were clustered closer together. The microbial structure between D0 and D14 of LW stud boars showed significant differences (PERMANOVA, *p <* 0.001), and although most of the samples overlapped, some samples were distributed outside the cluster. The clustering of samples in LAN stud boars was similar to that in LW, and there were significant differences in the clustering of samples fed with amoxicillin at different time points (PERMANOVA, *p <* 0.001). The D7 sample was clustered separately and showed significant differences without overlapping with the D0 and D14 samples (PERMANOVA, *p <* 0.001). Some D0 and D14 samples overlapped, and many samples were dispersed outside (Figure 2C). In DUR stud boars, there were significant differences in the microbial structure of the samples across different time points of amoxicillin feeding (PERMANOVA, *p <* 0.001). We found that D0 samples did not overlap with D7 and D14 samples, whereas some sample clustering overlapped between D7 and D14 (Figure 2D).

**Figure 2:**
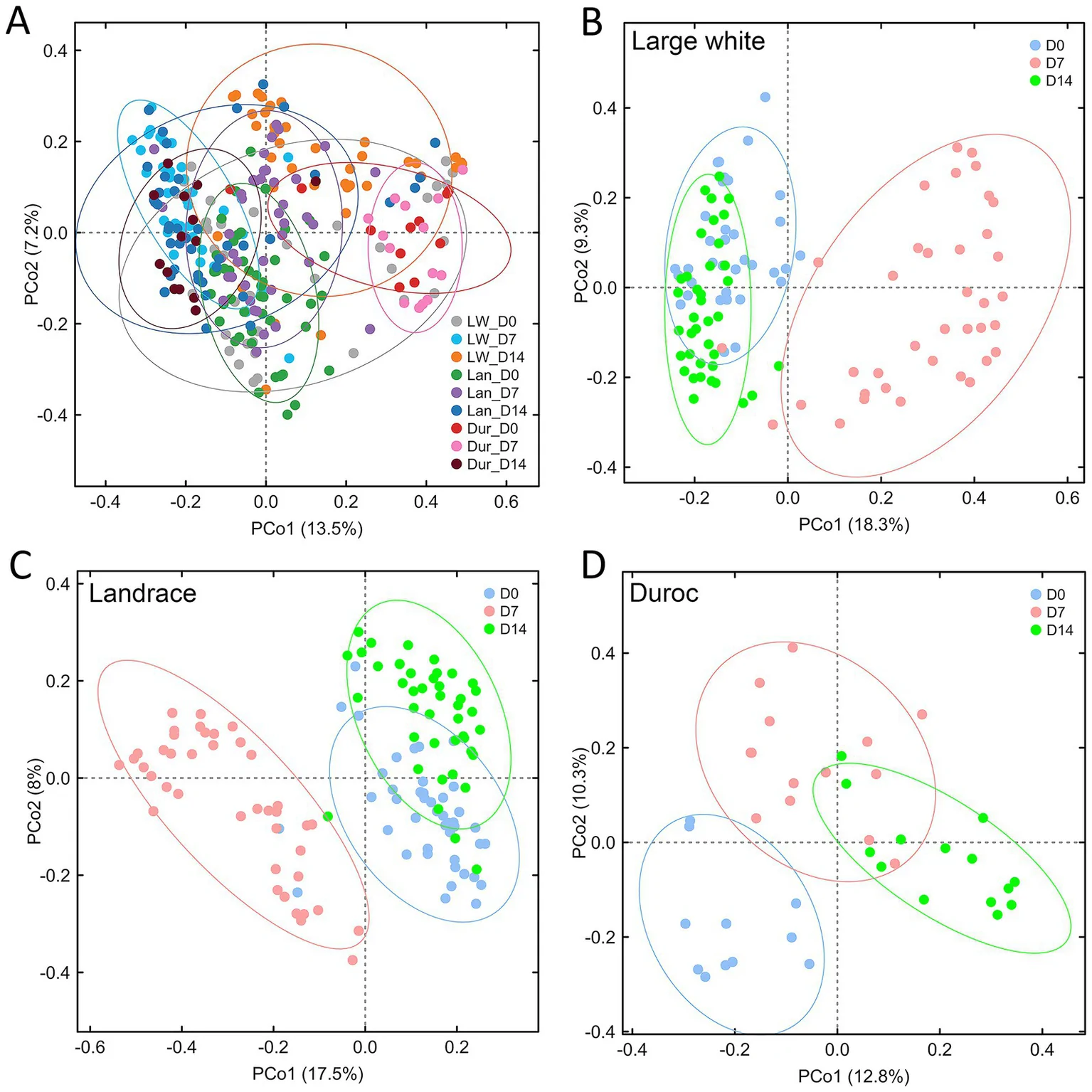
Structure of the gut microbiota of three breeds of stud boars. **(A)** PCoA of gut microbiota based on Bray-Curtis distance for all stud boars. **(B)** PCoA of gut microbiota for LW stud boars among D0, D7, and D14. **(C)** PCoA of gut microbiota for LAN stud boars among D0, D7, and D14. **(D)** PCoA of gut microbiota for DUR stud boars at D0, D7, and D14. PCoA, principal coordinate analysis.

To detect the effects of amoxicillin feeding on the structure of gut microbiota in stud boars of different breeds, we analyzed the beta diversity using the Bray–Curtis dissimilarity metric in PCoA. Before amoxicillin feeding (D0), there were significant differences in the microbial structure of the samples among the three breeds of stud boars (PERMANOVA, *p <* 0.001) (Figure 3A). DUR samples were clearly separated along PCo2, whereas LW and LAN partially overlapped. During amoxicillin feeding (D7), LW and DUR samples largely separated, but both overlapped with the LAN sample (Figure 3B). There were significant differences in the microbial structure of the samples among different breeds of stud boars in D7 (PERMANOVA, *p <* 0.001), with significant differences between each breed (PERMANOVA, *p <* 0.001). After amoxicillin feeding (D14), the clustering of samples was similar to that before amoxicillin feeding (Figure 3C). There were significant differences in the clustering of samples among the three breeds of stud boars (PERMANOVA, *p <* 0.001).

**Figure 3:**
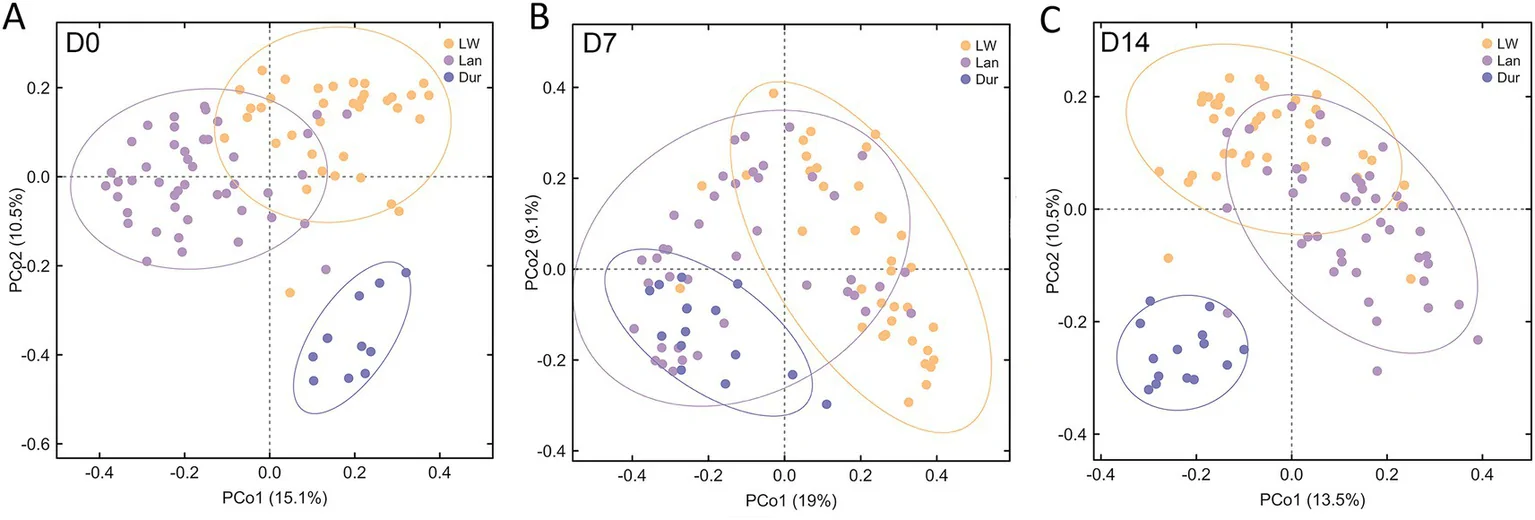
Gut microbiota structure at different time points of amoxicillin administration. **(A)** PCoA of gut microbiota at D0 among three breeds of stud boars. **(B)** PCoA of gut microbiota at D7 among three breeds of stud boars. **(C)** PCoA of gut microbiota at D14 among three breeds of stud boars.

### 3.4 Composition of microbial community in stud boars fed amoxicillin

A total of 19 bacterial phyla were identified in all samples, of which 12 had a relative abundance of more than 0.05% (Supplementary Figure S1). The predominant phyla across all breeds were *Firmicutes* (73.35%), *Bacteroidota* (14.45%), *Actinobacteriota* (6.28%), *Spirochaetota* (2.39%), *Proteobacteria* (2.31%), and other phyla. The relative abundance of *Actinobacteriota* and *Proteobacteria* increased in the three breeds of stud boars during amoxicillin feeding and decreased after amoxicillin withdrawal. At the genus level, a total of 327 genera were identified in all samples, among which 88 genera exhibited relative abundances exceeding 0.1% (Figure 4A). In the microbial community, *Lactobacillus* (8.77%), *Streptococcus* (7.09%), *Prevotella* (4.39%), *Clostridium_sensu_stricto_1* (3.85%), *Bacillus* (2.79%), *Bifidobacterium* (2.77%), *UCG-002* (2.26%), *Treponema* (2.15%), *Blautia* (2.06%), and *Veillonella* (2.06%) were the most dominant genera in all samples. In LW stud boars, the most dominant genera were *Lactobacillus* (11.87%), *Streptococcus* (10.87%), *Veillonella* (4.04%), *Prevotella* (3.62%), and *Bacillus* (3.6%). The relative abundance of *Lactobacillus* (17.2%), *Streptococcus* (16.67%), *Veillonella* (12.05%), and *Prevotella* (5.39%) increased during amoxicillin feeding in LW, whereas *Bacillus* (1.89%) decreased (Figure 4B; Supplementary Table S2). In LAN stud boars, *Lactobacillus* (6.15%), *Clostridium_sensu_stricto_1* (6.9%), *Streptococcus* (5.84%), *Treponema* (3.75%), *Bifidobacterium* (3.57%), and *Prevotella* (3.36%) were the predominant genera. When LAN stud boars were fed amoxicillin, the relative abundance of *Lactobacillus* (5.86%), *Streptococcus* (9.52%), *Bifidobacterium* (10.56%), and *Prevotella* (5.05%) increased, whereas *Clostridium_sensu_stricto_1* (0.43%) and *Treponema* (2.56%) decreased (Figure 4C; Supplementary Table S3). In DUR stud boars, the predominant genera were *Lactobacillus* (8.65%), *Prevotella* (9.94%), *Blautia* (4.96%), *Bacillus* (3.13%), and *Catenibacterium* (2.63%). The relative abundance of *Lactobacillus* (6.71%), *Bacillus* (2.91%), and *Catenibacterium* (5.40%) increased during amoxicillin feeding, and *Prevotella* (10.28%) and *Blautia* (3.28%) decreased (Figure 4D; Supplementary Table S4).

**Figure 4:**
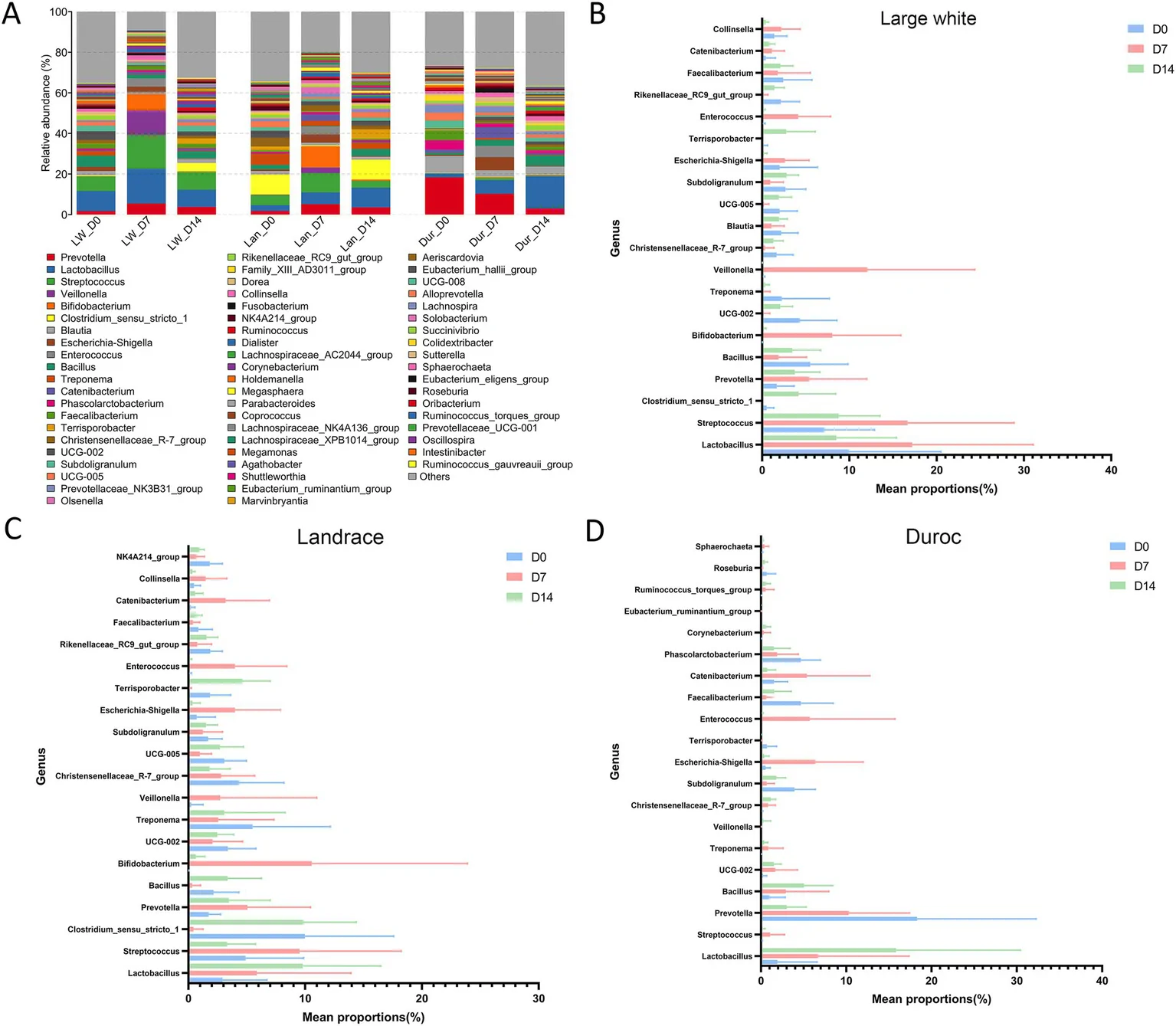
Composition of gut microbiota at the genus level. **(A)** The relative abundance of bacterial genera across different groups. Dominant genera of LW **(B)**, LAN **(C)**, and DUR **(D)** stud boars among D0, D7, and D14.

### 3.5 Differences in microbial community at different time points of amoxicillin feeding

To explore the differences in microbial community during amoxicillin feeding in stud boars, we analyzed the relative abundance of bacterial genera among the different time points of amoxicillin feeding. In LW, there were 85 bacterial genera with significant differences among the three time points of amoxicillin feeding (Supplementary Table S1). We further analyzed the differences in bacterial genera between each pair of time points of amoxicillin feeding. Between D0 and D7 of LW, 63 bacterial genera showed significant differences. Feeding amoxicillin significantly increased the relative abundance of *Lactobacillus*, *Streptococcus*, *Veillonella*, *Bifidobacterium*, *Prevotella*, *Enterococcus*, *Dialister*, *Corynebacterium*, *Catenibacterium*, etc., compared to D0. The relative abundance of *Bacillus*, *UCG-002*, *Subdoligranulum*, *Blautia,* and *Treponema* decreased significantly during amoxicillin feeding (Figure 5A; Supplementary Table S1). There were 59 bacterial genera that differed significantly between D7 and D14 in LW stud boars. After stopping amoxicillin feeding (D14), it was found that the relative abundance of *Clostridium_sensu_stricto_1*, *Subdoligranulum*, *Blautia*, *Terrisporobacter*, *UCG-002*, *UCG-005,* and *Ruminococcus* significantly increased compared with D7, whereas the relative abundance of *Lactobacillus*, *Streptococcus*, *Veillonella*, *Bifidobacterium,* and *Enterococcus* significantly decreased (Figure 5B; Supplementary Table S1). Between D0 and D14 of LW, there were 32 bacterial genera with significant differences. The relative abundance of *UCG-002*, *Escherichia–Shigella*, *Collinsella*, and *Phascolarctobacterium* was significantly higher in D0 than in D14. The relative abundance of *Prevotella*, *Clostridium_sensu_stricto_1*, *Terrisporobacter*, *Ruminococcus*, *Corynebacterium,* and *Dialister* in D14 was significantly higher than that in D0 (Figure 5C; Supplementary Table S1).

**Figure 5:**
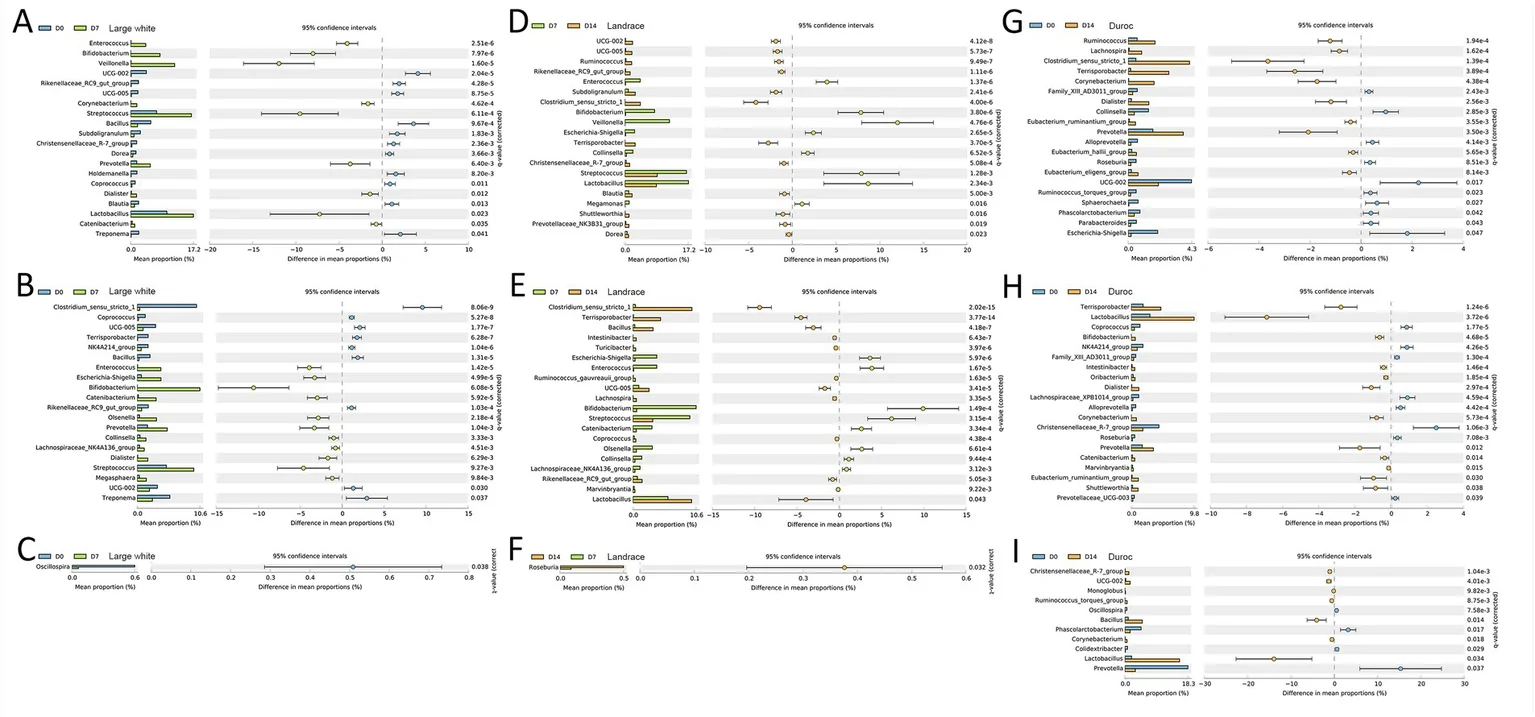
Differences in gut microbiota at the genus level. Significant differences in genera between D0 and D7 in LW **(A)**, LAN **(B)**, and DUR **(C)** stud boars. Significant differences in genera between D7 and D14 in LW **(D)**, LAN **(E)**, and DUR **(F)** stud boars. Significant differences in genera between D0 and D14 in LW **(G)**, LAN **(H)**, and DUR **(I)** stud boars. Significant differences between groups were tested by Welch’s *t-*test and Benjamini-Hochberg FDR correction.

In LAN stud boars, there were 75 bacterial genera with significant differences among the three time points of amoxicillin feeding (Supplementary Table S3). We identified 49 genera with significant differences between D0 and D7, and the relative abundance of *Clostridium_sensu_stricto_1*, *Treponema*, *UCG-002,* and *UCG-005* was higher in D0 than in D7, whereas *Streptococcus*, *Bifidobacterium*, *Prevotella*, *Escherichia–Shigella,* and *Enterococcus* were higher in D7 compared to D0 (Figure 5D; Supplementary Table S3). Between D7 and D14 of LAN, there were 34 genera that showed significant differences. After stopping amoxicillin feeding (D14), the relative abundance of *Lactobacillus*, *Clostridium_sensu_stricto_1*, *Terrisporobacter*, *UCG-005,* and *Bacillus* was significantly higher than that at D7, whereas the relative abundance of *Streptococcus*, *Bifidobacterium*, *Escherichia–Shigella*, *Enterococcus,* and *Olsenella* was significantly decreased (Figure 5E; Supplementary Table S3). Between D0 and D14 of LAN, there were 33 genera with significant differences. The relative abundance of *Christensenellaceae_R-7_group*, *NK4A214_group*, *Coprococcus, and Lachnospiraceae_XPB1014_group* was higher in D0 than in D14, whereas *Lactobacillus*, *Terrisporobacter*, *Prevotella*, *Dialister,* and *Shuttleworthia* were higher in D14 compared to D0 (Figure 5F; Supplementary Table S3).

In DUR stud boars, there were 33 bacterial genera with significant differences among the three time points of amoxicillin feeding (Supplementary Table S4). Only *Oscillospira* showed significant differences between D0 and D7, and the relative abundance of *Oscillospira* was higher in D0 than in D7 (Figure 5G; Supplementary Table S4). Similarly, we only found significant differences in *Roseburia* between D7 and D14, and the relative abundance of *Roseburia* was higher in D14 than in D7 (Figure 5H; Supplementary Table S4). There were 11 genera that were significantly different between D0 and D14. The relative abundance of *Prevotella*, *Phascolarctobacterium*, *Colidextribacter,* and *Oscillospira* was higher in D0 compared to D14, although *Lactobacillus*, *Bacillus*, *UCG-002*, and *Christensenellaceae_R-7_group* were higher in D14 than in D0 (Figure 5I; Supplementary Table S4).

Across all three breeds, no high-abundance core genera were completely undetectable at D7 during amoxicillin exposure. Most of the observed shifts in bacterial community composition represented changes in relative abundance rather than the complete loss of taxa. Nevertheless, several low-abundance genera were not detected in a subset of individual D7 samples, indicating that amoxicillin could drive sample-level dropout of rare taxa, even though these taxa persisted across the full cohort. Because the present study relies on 16S rRNA amplicon sequencing of relative abundances, we cannot definitively rule out true absolute-abundance extirpation for some low-frequency microbes.

### 3.6 Predicted functional capacities of microbiota

We predicted the Kyoto Encyclopedia of Genes and Genomes (KEGG) pathways (31) of gut microbiota in three breeds of stud boars fed amoxicillin using 16S rRNA data. To investigate the differences in KEGG pathways of the gut microbial community at different time points of amoxicillin feeding, linear discriminant analysis effect size (LEfSe) analysis of KEGG pathways was performed. In LW stud boars, there were 266 KEGG Orthologs (KOs) with significant differences across different time points of amoxicillin feeding (Figure 6A; Supplementary Table S5). A total of 126 KOs were significant markers in D0 of LW, of which the top 10 KOs were mainly associated with signal transduction, global and overview maps, energy metabolism, amino acid metabolism, carbohydrate metabolism, lipid metabolism, and drug resistance (antimicrobial). During amoxicillin feeding (D7) in LW, 77 KOs were significantly enriched, mostly in relation to global and overview maps, membrane transport, glycan biosynthesis and metabolism, carbohydrate metabolism, cellular community (prokaryotes), metabolism of cofactors and vitamins, and drug resistance: antimicrobial. After stopping amoxicillin feeding (D14) in LW, 63 KOs showing higher abundances were mostly correlated with the global and overview maps, membrane transport, glycan biosynthesis and metabolism, carbohydrate metabolism, cellular community (prokaryotes), metabolism of cofactors and vitamins, and drug resistance: antimicrobial.

**Figure 6:**
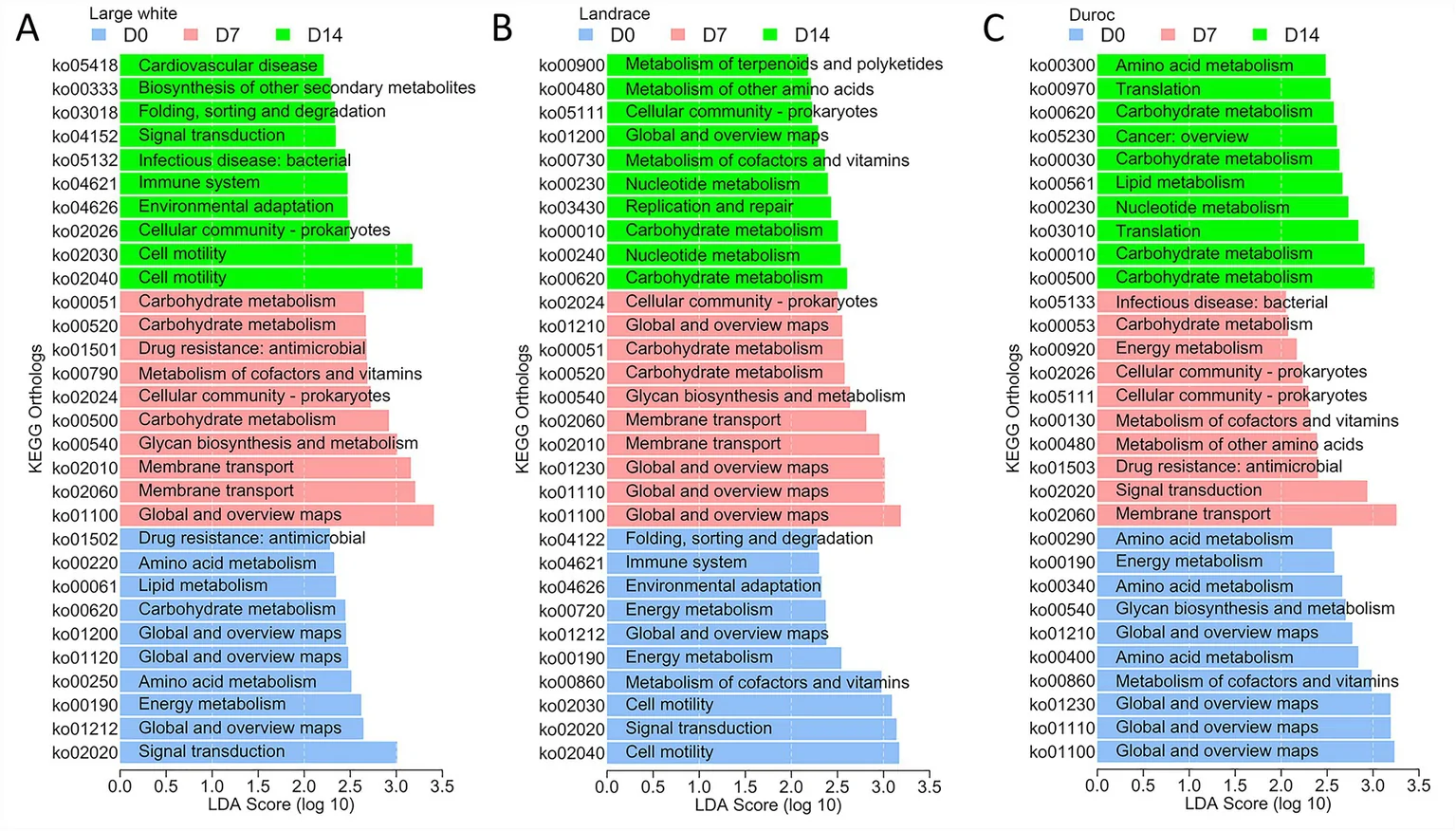
Predicted functional capacities of KEGG Ortholog pathways in three breeds of stud boars among D0, D7, and D14. **(A)** LW stud boars. **(B)** LAN stud boars. **(C)** DUR stud boars. Significantly different KEGG Ortholog pathways are shown with an LDA threshold > 2.0. KEGG, Kyoto Encyclopedia of Genes and Genomes; LDA, linear discriminant analysis.

In LAN stud boars, there were 246 KOs with significant differences across different time points of amoxicillin feeding (Figure 6B; Supplementary Table S6). A total of 92 KOs were significantly enriched in D0 of LAN, of which the top 10 KOs were mainly linked to folding, sorting, and degradation; the immune system; environmental adaptation; energy metabolism; global and overview maps; metabolism of cofactors and vitamins; cell motility; and signal transduction. Eighty-five KOs were significantly enriched during amoxicillin feeding (D7) in LAN, primarily in association with global and overview maps, membrane transport, glycan biosynthesis and metabolism, cellular community (prokaryotes), and carbohydrate metabolism. After stopping amoxicillin feeding (D14) in LAN, 69 KOs exhibiting elevated abundances were predominantly associated with carbohydrate metabolism, metabolism of terpenoids and polyketides, nucleotide metabolism, replication and repair, metabolism of cofactors and vitamins, global and overview maps, cellular communities (prokaryotes), and metabolism of other amino acids.

There were 139 KOs in DUR stud boars with significant differences among amoxicillin feeding times (Figure 6C; Supplementary Table S7). On day 0, 46 KOs were significantly enriched and correlated with global and overview maps, metabolism of cofactors and vitamins, amino acid metabolism, glycan biosynthesis and metabolism, and energy metabolism. Fifty KOs were significantly enriched during amoxicillin feeding (D7) in DUR, and their pathways were related to carbohydrate metabolism, cellular community (prokaryotes), membrane transport, signal transduction, drug resistance (antimicrobial), metabolism of other amino acids, metabolism of cofactors and vitamins, energy metabolism, and infectious disease (bacterial). The 43 KOs with greater abundances following amoxicillin feeding (D14) in DUR were primarily associated with carbohydrate metabolism, translation, nucleotide metabolism, lipid metabolism, cancer (overview), and amino acid metabolism.

## 4 Discussion

Amoxicillin is currently recognized as an exceptionally valuable antimicrobial in both human and veterinary medicine, and it is categorized as a critically important antimicrobial by the World Health Organization (32). Amoxicillin is widely used as an antibiotic in pig farming (33–35). Amoxicillin serves as the primary antimicrobial agent administered during the weaning phase, primarily to combat infections caused by enterotoxigenic *Escherichia coli* (ETEC) and *Streptococcus suis* (5, 36). Oral administration of amoxicillin reduces the alpha diversity of intestinal microbiota and affects the composition of the microbial community (36, 37). Modern industrial agriculture has shown growing concern over the gut microbiome and intestinal health (38). Therefore, there is an urgent need for studies on the structural and functional changes in the pig gut microbiota following the use of antibiotics like amoxicillin. However, studies on the effects of short-term amoxicillin administration on porcine gut microbial communities and their post-dosing recovery remain limited. In this study, fecal samples were collected from LW, LAN, and DUR at three distinct time points: 3 days prior to amoxicillin administration, during 4 consecutive days of administration, and 7 days post-withdrawal. Using 16S rRNA sequencing, we systematically characterized the dynamic shifts in gut microbiota composition and structure before, during, and after amoxicillin exposure. This study provides a reference for the rational use of amoxicillin in livestock production.

This study revealed that in both LW and LAN stud boars, the Sobs index of alpha-diversity significantly decreased following amoxicillin administration and rebounded after drug withdrawal. Concomitantly, the Shannon index of alpha-diversity showed a parallel trend. Studies have demonstrated that the effects of antibiotics on the gut microbiota subside shortly after treatment cessation, with the overall microbial composition rapidly reverting to a near initial state (17). However, in DUR pigs, the Sobs and Shannon alpha-diversity indices trended upward following administration and showed a recovery trend after drug withdrawal, though the differences were not statistically significant. These non-significant alpha-diversity changes in DUR should be interpreted cautiously, given the smaller sample size of this breed group, which may have reduced the statistical power for detecting true biological differences. The trend of alpha-diversity observed in DUR stud boars following amoxicillin administration aligns with findings on intestinal microbiota changes in mice after antibiotic exposure (39). Collectively, these results highlight breed-specific responses of gut microbiota diversity to antibiotic exposure, with DUR stud boars exhibiting a divergent trend that may reflect genetic or strain-dependent resilience. This divergence, although statistically non-significant, aligns with emerging evidence of interspecies variation in microbiota recovery kinetics (40).

This study revealed significant differences in microbial structure among the three breeds of stud boars’ samples before amoxicillin feeding (D0). Notably, PCoA revealed that samples of all DUR stud boars were completely segregated from those of the other two breeds both before and after amoxicillin administration. The microbial structural differences between DUR stud boars and the other two breeds may be attributed to host genetics, which is widely recognized as a key determinant of intestinal microbiota diversity (41). Furthermore, microbial communities have been shown to exhibit breed-specific variations in pigs (42). Throughout the study, *Lactobacillus* and *Streptococcus* were the consistent dominant genera in LW and LAN stud boars. By contrast, DUR stud boars exhibited distinct dominance of *Prevotella* and *Lactobacillus*. Another study found the microbiota of LW pigs differed significantly from that of DUR pigs, suggesting distinct breed-specific characteristics (41). Moreover, studies have demonstrated that genetically related individuals exhibit more similar gut microbiota compositions than unrelated counterparts (43).

Following amoxicillin administration to the three pig breeds in this study, the relative abundances of most intestinal microbiota genera either decreased or increased, with levels reverting to near-baseline 7 days post-withdrawal. A similar pattern has been observed in the intestinal microbiota of humans treated with ciprofloxacin. One week after ceasing ciprofloxacin administration, the microbiota composition begins to revert to its initial state, though this recovery is often incomplete (17). Although the overall diversity and composition of the gut microbiota appear to recover rapidly, certain specific alterations may persist for extended periods (44).

Functional prediction using PICRUSt2 indicated that taxonomic remodeling was accompanied by shifts in inferred KEGG pathways (45, 46). At D7, the gut microbiota of LW and LAN stud boars were enriched for carbohydrate metabolism, glycan biosynthesis and metabolism, membrane transport, and cellular community functions. This functional profile is biologically consistent with the concurrent expansion of *Lactobacillus, Streptococcus*, and *Bifidobacterium*. In pigs, *Lactobacillus* is a major contributor to the fermentation of fructans and simple sugars (47). Therefore, enrichment of carbohydrate- and glycan-related KEGG pathways is the expected metabolic consequence of the blooming lactic acid bacteria when more β-lactam-sensitive anaerobes decline. Enrichment of membrane-transport functions is consistent with nutrient scavenging and efflux-related stress responses. Gut bacteria commonly upregulate metabolite-transport and multidrug-resistance pathways when exposed to orally administered drugs (48). Antimicrobial resistance pathways were enriched at D7 in LW and DUR and remained among the differential functions at D14 in LW, suggesting that even a short course of amoxicillin can leave a resistance-related functional imprint after partial taxonomic recovery. A similar dissociation between relatively rapid compositional rebound and longer-lasting metabolic or resistome disturbance has been reported after short-term antibiotic exposure (14, 46) and after amoxicillin treatment in pigs (36, 37). By contrast, D0 communities were more strongly associated with energy, amino-acid, and lipid metabolism and with signal transduction, indicating a broader metabolic repertoire that contracted during treatment. After withdrawal (D14), carbohydrate, nucleotide, and cofactor metabolism again became prominent, coinciding with the rebound of several genera and suggesting partial restoration of fermentative metabolism. Fewer KEGG orthologs differed among time points in DUR, paralleling the weaker taxonomic response in this breed, although resistance- and transport-related functions were still enriched at D7. Because these profiles are inferred from 16S rRNA data rather than measured gene content, they should be interpreted as hypothesis-generating; shotgun metagenomics will be required to confirm the predicted metabolic and resistome changes (30).

This study represents the first systematic characterization of gut microbiota dynamics and recovery following short-term amoxicillin exposure in three breeds of stud boars; nonetheless, several experimental limitations exist. First, no parallel untreated negative control was established. Despite using pre-treatment (Day 0) samples as self-controls for each boar, missing a non-medicated cohort could mask spontaneous time-dependent microbiome changes linked to age, environment, and husbandry conditions. Second, ethical concerns and the high economic value of stud boars restricted sampling to feces, which only represent luminal microbes of the distal gut and cannot reflect unique microbial microhabitats in the small intestine, cecum, and intestinal mucosa. Third, the sample size of Duroc boars in the present study was relatively limited. The complete pedigree information was unavailable, so we could not quantitatively assess the potential litter-origin effect on microbiome shifts upon amoxicillin exposure. To address these limitations, future studies should incorporate a parallel, unmedicated control group and utilize advanced non-invasive multi-segment sampling methodologies to comprehensively map the microbial landscape across diverse intestinal microhabitats. Nevertheless, this study provides crucial baseline insights and a valuable scientific reference for optimizing antibiotic stewardship and understanding breed-specific microbial resilience in swine production.

## 5 Conclusion

This study explored gut microbiota dynamics in LW, LAN, and DUR boars before, during, and after amoxicillin administration. Amoxicillin reduced alpha-diversity in LW and LAN at D7, with partial recovery by D14, whereas DUR showed no significant changes. PCoA revealed distinct microbial clustering at D7. *Lactobacillus* and *Streptococcus* dominated LW and LAN, with abundances rising during treatment and falling post-withdrawal; DUR featured *Prevotella* and *Lactobacillus*. KEGG pathways indicated altered metabolism and drug resistance. Findings highlight breed-specific responses, supporting optimized antibiotic use in pig farming to reduce resistance risks.

## Data Availability

The datasets presented in this study can be found in online repositories. The names of the repository/repositories and accession number(s) can be found below: Figshare, https://figshare.com/articles/dataset/16S_rRNA_sequencing_data_of_fecal_samples_from_three_breeds_of_boars/29382245.
