# Supplementary material for: Dynamic changes in gut microbiota of three breeds of stud boars pre- and post-amoxicillin administration
Source: Front Vet Sci. 2026 Oct 1;13:1930671. doi: 10.3389/fvets.2026.1930671 (PMC13671024; doi:10.3389/fvets.2026.1930671)
Supplement: Supplementary file 1 [file Supplementary_file_1.DOCX]

Supplementary Material

Dynamic changes of gut microbiota in three breeds of stud boars pre- and post-amoxicillin administration

Liusheng Wang^1^, Huiting He^2^, Tao Huang^2^, and Zhelong Jiang^3*^

^1^Department of Emergency, Affiliated Hangzhou First People's Hospital, School of Medicine, Westlake University, Hangzhou, China

^2^Key Laboratory of Applied Technology on Green-Eco-Healthy Animal Husbandry of Zhejiang Province, College of Animal Science and Technology, College of Veterinary Medicine, Zhejiang A&F University, Hangzhou, China

^3^Department of Respiratory and Critical Medicine, Affiliated Hangzhou First People's Hospital, School of Medicine, Westlake University, Hangzhou, China

*** Correspondence:** Zhelong Jiang: [zhelongjiang2025@163.com](mailto: zhelongjiang2025@163.com )

# 1 Supplementary Figures and Tables

## 1.1 Supplementary Figures

**Supplementary Figure 1.** Composition of microbiota on phylum level.

## 1.2 Supplementary Tables

**Supplementary Table 1.** Summary of samples, sequencing data, and ASV data.

| **Breed** | **Group** | **Sample number** | **Sequence number** | **ASVs passed quality control** | **Annotated phylum** | **Annotated genus** |
| --- | --- | --- | --- | --- | --- | --- |
| large white | D0 | 36 | 57855.72 ± 15017.05 | 2876 | 17 | 261 |
|  | D7 | 37 | 60850.62 ± 8695.00 | 1723 | 14 | 260 |
|  | D14 | 38 | 58249.08 ± 9517.10 | 2745 | 15 | 256 |
| landrace | D0 | 45 | 59699.38 ± 7620.45 | 3972 | 18 | 281 |
|  | D7 | 41 | 58669.68 ± 6779.01 | 2961 | 15 | 291 |
|  | D14 | 43 | 66844.30 ± 16405.74 | 3437 | 18 | 265 |
| duroc | D0 | 11 | 62769.00 ± 14071.73 | 1066 | 11 | 163 |
|  | D7 | 14 | 63173.07 ± 12643.56 | 1733 | 12 | 244 |
|  | D14 | 14 | 50962.36 ± 7633.71 | 1627 | 13 | 202 |

**Supplementary Table 2.** The compensation of microbiota for different time points of LW stud boars at the genus level.

| **Genera** | **Relative abundance(%)** | | | | ***p*-value** | ***p*-value (corrected)** |
| --- | --- | --- | --- | --- | --- | --- |
|  | **ALL** | **D0** | **D7** | **D14** |  |  |
| *Lactobacillus* | 11.87 ± 11.35 | 9.93 ± 10.57 | 17.2 ± 13.85 | 8.52 ± 6.86 | 0.02 | 0.02 |
| *Streptococcus* | 10.87 ± 9.17 | 7.12 ± 5.74 | 16.67 ± 12.19 | 8.76 ± 4.74 | 1.47E-05 | 1.91E-05 |
| *Clostridium_sensu_stricto_1* | 1.6 ± 3.13 | 0.52 ± 0.81 | 0 ± 0.01 | 4.17 ± 4.25 | 1.21E-19 | 5.32E-18 |
| *Prevotella* | 3.62 ± 4.53 | 1.68 ± 1.99 | 5.39 ± 6.59 | 3.75 ± 2.86 | 6.41E-04 | 7.32E-04 |
| *Bacillus* | 3.6 ± 3.88 | 5.52 ± 4.32 | 1.89 ± 3.2 | 3.46 ± 3.25 | 3.01E-06 | 4.14E-06 |
| *Bifidobacterium* | 2.75 ± 5.86 | 0.01 ± 0.02 | 8.03 ± 7.86 | 0.19 ± 0.25 | 8.34E-20 | 7.34E-18 |
| *UCG-002* | 2.16 ± 3.08 | 4.31 ± 4.26 | 0.17 ± 0.71 | 2.05 ± 1.43 | 1.36E-14 | 1.33E-13 |
| *Treponema* | 0.89 ± 3.27 | 2.25 ± 5.46 | 0.14 ± 0.76 | 0.33 ± 0.49 | 2.42E-12 | 8.52E-12 |
| *Veillonella* | 4.04 ± 9.05 | 0.08 ± 0.23 | 12.05 ± 12.31 | 0 ± 0.02 | 2.34E-16 | 2.94E-15 |
| *Christensenellaceae_R-7_group* | 1.09 ± 1.48 | 1.65 ± 1.88 | 0.33 ± 1.01 | 1.29 ± 1.11 | 7.38E-10 | 1.55E-09 |
| *Blautia* | 1.72 ± 1.55 | 2.17 ± 1.92 | 1.05 ± 1.46 | 1.95 ± 0.94 | 8.69E-05 | 1.08E-04 |
| *UCG-005* | 1.37 ± 1.68 | 2 ± 2.03 | 0.21 ± 0.54 | 1.9 ± 1.48 | 1.48E-12 | 5.90E-12 |
| *Subdoligranulum* | 2.14 ± 1.95 | 2.68 ± 2.33 | 0.93 ± 1.49 | 2.8 ± 1.36 | 1.50E-07 | 2.54E-07 |
| *Escherichia-Shigella* | 1.59 ± 3.1 | 2.01 ± 4.33 | 2.61 ± 2.76 | 0.2 ± 0.35 | 6.00E-10 | 1.29E-09 |
| *Terrisporobacter* | 1 ± 2.33 | 0.17 ± 0.45 | 0 ± 0 | 2.76 ± 3.32 | 7.92E-18 | 1.74E-16 |
| *Enterococcus* | 1.47 ± 2.85 | 0.08 ± 0.27 | 4.14 ± 3.7 | 0.18 ± 0.19 | 2.79E-18 | 8.19E-17 |
| *Rikenellaceae_RC9_gut_group* | 1.24 ± 1.61 | 2.14 ± 2.12 | 0.18 ± 0.48 | 1.41 ± 1.12 | 2.42E-13 | 1.33E-12 |
| *Faecalibacterium* | 2.09 ± 2.96 | 2.41 ± 3.3 | 1.79 ± 3.71 | 2.08 ± 1.44 | 0.01 | 0.01 |
| *Catenibacterium* | 0.79 ± 1.13 | 0.42 ± 1.1 | 1.12 ± 1.44 | 0.81 ± 0.64 | 1.12E-05 | 1.47E-05 |
| *Collinsella* | 1.32 ± 1.67 | 1.38 ± 1.46 | 2.18 ± 2.18 | 0.41 ± 0.32 | 8.52E-07 | 1.34E-06 |
| *Dorea* | 1.01 ± 0.98 | 1.45 ± 1.28 | 0.6 ± 0.73 | 1 ± 0.68 | 2.74E-04 | 3.22E-04 |
| *Prevotellaceae_NK3B31_group* | 0.6 ± 1.28 | 0.47 ± 0.91 | 0.25 ± 0.62 | 1.07 ± 1.83 | 2.09E-06 | 3.17E-06 |
| *Ruminococcus* | 0.92 ± 1.14 | 0.61 ± 0.51 | 0.28 ± 0.7 | 1.82 ± 1.35 | 3.98E-13 | 1.94E-12 |
| *NK4A214_group* | 0.68 ± 0.93 | 1.06 ± 1.15 | 0.21 ± 0.84 | 0.8 ± 0.52 | 1.75E-12 | 6.70E-12 |
| *Dialister* | 1.09 ± 2.05 | 0.22 ± 0.46 | 1.63 ± 2.87 | 1.4 ± 1.79 | 9.20E-06 | 1.23E-05 |
| *Phascolarctobacterium* | 0.74 ± 1.14 | 0.8 ± 0.76 | 1.02 ± 1.71 | 0.41 ± 0.51 | 0.03 | 0.03 |
| *Olsenella* | 0.43 ± 0.6 | 0.33 ± 0.38 | 0.53 ± 0.85 | 0.44 ± 0.47 | 0.24 | 0.24 |
| *Corynebacterium* | 1.18 ± 1.98 | 0.03 ± 0.09 | 1.69 ± 2.2 | 1.76 ± 2.26 | 1.39E-12 | 5.81E-12 |
| *Coprococcus* | 0.73 ± 1.13 | 1.24 ± 1.6 | 0.34 ± 0.91 | 0.63 ± 0.38 | 1.34E-08 | 2.52E-08 |
| *Megasphaera* | 0.53 ± 1.37 | 0.1 ± 0.23 | 1.07 ± 2.18 | 0.41 ± 0.66 | 0.01 | 0.01 |
| *Holdemanella* | 0.81 ± 1.82 | 1.72 ± 2.95 | 0.15 ± 0.26 | 0.58 ± 0.51 | 2.65E-11 | 7.53E-11 |
| *Lachnospiraceae_NK4A136_group* | 0.3 ± 0.65 | 0.32 ± 0.79 | 0.27 ± 0.71 | 0.31 ± 0.45 | 1.56E-03 | 1.74E-03 |
| *Shuttleworthia* | 0.45 ± 1.51 | 0.16 ± 0.41 | 0.04 ± 0.18 | 1.12 ± 2.42 | 2.14E-09 | 4.29E-09 |
| *Agathobacter* | 0.69 ± 1.42 | 0.91 ± 1.56 | 0.02 ± 0.09 | 1.13 ± 1.72 | 7.31E-14 | 4.95E-13 |
| *Eubacterium_ruminantium_group* | 0.2 ± 0.45 | 0.08 ± 0.16 | 0.02 ± 0.07 | 0.49 ± 0.66 | 1.39E-14 | 1.22E-13 |
| *Oribacterium* | 0.45 ± 0.53 | 0.43 ± 0.59 | 0.27 ± 0.52 | 0.64 ± 0.41 | 2.55E-06 | 3.67E-06 |
| *Lachnospiraceae_XPB1014_group* | 0.17 ± 0.8 | 0.39 ± 1.31 | 0.04 ± 0.23 | 0.09 ± 0.36 | 4.20E-04 | 4.86E-04 |
| *Solobacterium* | 0.45 ± 0.59 | 0.58 ± 0.73 | 0.22 ± 0.56 | 0.55 ± 0.39 | 1.37E-07 | 2.36E-07 |
| *Family_XIII_AD3011_group* | 0.31 ± 0.37 | 0.62 ± 0.44 | 0.02 ± 0.11 | 0.3 ± 0.17 | 3.44E-17 | 6.05E-16 |
| *Eubacterium_hallii_group* | 0.35 ± 0.41 | 0.25 ± 0.31 | 0.25 ± 0.39 | 0.56 ± 0.46 | 2.38E-06 | 3.49E-06 |
| *Alloprevotella* | 0.35 ± 0.63 | 0.63 ± 0.7 | 0.24 ± 0.75 | 0.18 ± 0.24 | 2.34E-06 | 3.49E-06 |
| *Marvinbryantia* | 0.45 ± 2.43 | 0.19 ± 0.21 | 0.07 ± 0.16 | 1.07 ± 4.12 | 3.65E-13 | 1.89E-12 |
| *Ruminococcus_gauvreauii_group* | 0.34 ± 0.56 | 0.43 ± 0.62 | 0.03 ± 0.07 | 0.55 ± 0.65 | 1.76E-12 | 6.44E-12 |
| *Ruminococcus_torques_group* | 0.41 ± 0.63 | 0.54 ± 0.75 | 0.54 ± 0.73 | 0.17 ± 0.15 | 0.1 | 0.1 |
| *Lachnospiraceae_AC2044_group* | 0.03 ± 0.15 | 0.09 ± 0.26 | 0 ± 0 | 0.02 ± 0.07 | 1.38E-04 | 1.66E-04 |
| *Fusobacterium* | 0.37 ± 1.75 | 0.31 ± 1.18 | 0.82 ± 2.77 | 0 ± 0 | 1.21E-09 | 2.47E-09 |
| *Bacteroides* | 0.24 ± 1.56 | 0.2 ± 0.5 | 0.49 ± 2.65 | 0.03 ± 0.06 | 1.05E-03 | 1.18E-03 |
| *Parabacteroides* | 0.26 ± 0.56 | 0.55 ± 0.89 | 0.06 ± 0.21 | 0.17 ± 0.13 | 4.23E-10 | 9.31E-10 |
| *Lachnospira* | 0.36 ± 0.71 | 0.07 ± 0.11 | 0.09 ± 0.18 | 0.92 ± 0.99 | 4.45E-11 | 1.19E-10 |
| *Roseburia* | 0.25 ± 0.42 | 0.53 ± 0.6 | 0.03 ± 0.12 | 0.18 ± 0.23 | 3.84E-11 | 1.06E-10 |
| *Prevotellaceae_UCG-003* | 0.19 ± 0.49 | 0.35 ± 0.8 | 0.08 ± 0.14 | 0.15 ± 0.25 | 3.56E-05 | 4.48E-05 |
| *Erysipelotrichaceae_UCG-006* | 0.31 ± 0.53 | 0.32 ± 0.42 | 0.18 ± 0.49 | 0.43 ± 0.63 | 2.89E-07 | 4.63E-07 |
| *Eubacterium_eligens_group* | 0.32 ± 0.56 | 0.22 ± 0.23 | 0.07 ± 0.16 | 0.67 ± 0.81 | 1.27E-11 | 3.71E-11 |
| *Prevotellaceae_UCG-001* | 0.05 ± 0.13 | 0.06 ± 0.13 | 0.02 ± 0.14 | 0.08 ± 0.12 | 1.83E-06 | 2.82E-06 |
| *Sphaerochaeta* | 0.24 ± 0.81 | 0.67 ± 1.33 | 0.01 ± 0.04 | 0.04 ± 0.05 | 4.90E-11 | 1.27E-10 |
| *Megamonas* | 0.4 ± 1.52 | 0.03 ± 0.16 | 1.15 ± 2.49 | 0.03 ± 0.06 | 1.23E-11 | 3.74E-11 |
| *Fournierella* | 0.21 ± 0.35 | 0.35 ± 0.54 | 0.08 ± 0.15 | 0.21 ± 0.18 | 2.97E-06 | 4.15E-06 |
| *Butyricicoccus* | 0.22 ± 0.29 | 0.3 ± 0.36 | 0.14 ± 0.25 | 0.23 ± 0.23 | 4.55E-03 | 4.95E-03 |
| *Eubacterium_siraeum_group* | 0.13 ± 0.19 | 0.17 ± 0.24 | 0.01 ± 0.03 | 0.2 ± 0.16 | 1.46E-13 | 8.59E-13 |
| *UCG-008* | 0.06 ± 0.11 | 0.02 ± 0.05 | 0.01 ± 0.01 | 0.14 ± 0.15 | 4.80E-13 | 2.22E-12 |
| *Succinivibrio* | 0.28 ± 0.95 | 0.01 ± 0.02 | 0.81 ± 1.53 | 0.02 ± 0.03 | 7.91E-08 | 1.39E-07 |
| *Turicibacter* | 0.06 ± 0.17 | 0.05 ± 0.21 | 0 ± 0 | 0.13 ± 0.19 | 2.92E-12 | 9.52E-12 |
| *Acidaminococcus* | 0.18 ± 0.47 | 0.04 ± 0.1 | 0.42 ± 0.74 | 0.08 ± 0.15 | 0.01 | 0.01 |
| *Intestinibacter* | 0.1 ± 0.21 | 0.01 ± 0.03 | 0 ± 0 | 0.28 ± 0.28 | 1.37E-13 | 8.60E-13 |
| *Eubacterium_nodatum_group* | 0.1 ± 0.23 | 0.21 ± 0.35 | 0.01 ± 0.02 | 0.1 ± 0.14 | 3.61E-10 | 8.15E-10 |
| *Aeriscardovia* | 0.36 ± 1.05 | 0 ± 0 | 1.07 ± 1.61 | 0 ± 0 | 2.56E-14 | 1.88E-13 |
| *Campylobacter* | 0.2 ± 0.56 | 0.43 ± 0.82 | 0.01 ± 0.06 | 0.16 ± 0.44 | 2.75E-12 | 9.30E-12 |
| *Desulfovibrio* | 0.12 ± 0.33 | 0.03 ± 0.07 | 0.1 ± 0.43 | 0.23 ± 0.35 | 7.28E-06 | 9.86E-06 |
| *Oscillospira* | 0.11 ± 0.23 | 0.04 ± 0.09 | 0 ± 0.01 | 0.28 ± 0.32 | 1.06E-16 | 1.55E-15 |
| *Colidextribacter* | 0.1 ± 0.19 | 0.16 ± 0.3 | 0.04 ± 0.08 | 0.11 ± 0.08 | 1.94E-07 | 3.22E-07 |
| *Lachnospiraceae_FCS020_group* | 0.14 ± 0.15 | 0.13 ± 0.09 | 0.14 ± 0.22 | 0.17 ± 0.1 | 1.92E-03 | 2.11E-03 |
| *Mitsuokella* | 0.14 ± 0.42 | 0.02 ± 0.08 | 0.28 ± 0.68 | 0.11 ± 0.19 | 2.84E-06 | 4.03E-06 |
| *Monoglobus* | 0.11 ± 0.15 | 0.13 ± 0.18 | 0.02 ± 0.07 | 0.18 ± 0.14 | 4.12E-12 | 1.29E-11 |
| *Lachnoclostridium* | 0.08 ± 0.11 | 0.07 ± 0.1 | 0.02 ± 0.04 | 0.13 ± 0.13 | 2.46E-07 | 4.01E-07 |
| *dgA-11_gut_group* | 0.13 ± 0.44 | 0.36 ± 0.72 | 0 ± 0.01 | 0.04 ± 0.06 | 1.63E-10 | 3.87E-10 |
| *Candidatus_Soleaferrea* | 0.07 ± 0.15 | 0.15 ± 0.24 | 0.02 ± 0.04 | 0.05 ± 0.06 | 2.21E-04 | 2.63E-04 |
| *Negativibacillus* | 0.08 ± 0.12 | 0.07 ± 0.08 | 0.01 ± 0.04 | 0.16 ± 0.15 | 8.26E-11 | 2.08E-10 |
| *Mogibacterium* | 0.08 ± 0.11 | 0.08 ± 0.08 | 0.03 ± 0.06 | 0.14 ± 0.13 | 7.37E-09 | 1.41E-08 |
| *Sutterella* | 0.25 ± 0.7 | 0.02 ± 0.04 | 0.74 ± 1.07 | 0.01 ± 0.03 | 2.46E-14 | 1.97E-13 |
| *Staphylococcus* | 0.13 ± 0.2 | 0.05 ± 0.12 | 0.17 ± 0.23 | 0.17 ± 0.22 | 1.02E-04 | 1.25E-04 |
| *Fusicatenibacter* | 0.13 ± 0.18 | 0.17 ± 0.23 | 0.03 ± 0.06 | 0.19 ± 0.18 | 1.40E-08 | 2.57E-08 |
| *Lachnospiraceae_NK3A20_group* | 0.14 ± 0.5 | 0.05 ± 0.08 | 0.01 ± 0.03 | 0.35 ± 0.81 | 3.50E-09 | 6.84E-09 |
| *Clostridium_sensu_stricto_6* | 0.12 ± 0.29 | 0.15 ± 0.31 | 0 ± 0.01 | 0.2 ± 0.37 | 1.19E-12 | 5.24E-12 |
| *UCG-009* | 0.08 ± 0.13 | 0.11 ± 0.2 | 0.02 ± 0.07 | 0.1 ± 0.07 | 1.21E-10 | 2.96E-10 |
| *Syntrophococcus* | 0.15 ± 0.8 | 0.12 ± 0.25 | 0.01 ± 0.02 | 0.32 ± 1.34 | 2.19E-05 | 2.80E-05 |
| *Anaerostipes* | 0.09 ± 0.19 | 0.05 ± 0.08 | 0 ± 0.01 | 0.2 ± 0.28 | 3.35E-10 | 7.77E-10 |
| *Enterorhabdus* | 0.08 ± 0.11 | 0.09 ± 0.14 | 0.03 ± 0.07 | 0.12 ± 0.11 | 6.09E-08 | 1.09E-07 |
| *Others* | 21.9 ± 12.57 | 30.9 ± 8.37 | 6.89 ± 5.71 | 27.98 ± 5.41 | 7.42E-16 | 8.16E-15 |

**Supplementary Table 3.** The compensation of microbiota for different time points of LAN stud boars at the genus level.

| **Genera** | **Relative abundance(%)** | | | | ***p*-value** | ***p*-value (corrected)** |
| --- | --- | --- | --- | --- | --- | --- |
|  | **ALL** | **D0** | **D7** | **D14** |  |  |
| *Lactobacillus* | 6.15 ±6.93 | 2.92 ± 3.8 | 5.86 ± 8.06 | 9.8 ± 6.65 | 9.51E-07 | 2.15E-06 |
| *Streptococcus* | 5.84 ±6.39 | 4.9 ± 4.97 | 9.52 ± 8.71 | 3.32 ± 2.41 | 2.19E-03 | 3.12E-03 |
| *Clostridium_sensu_stricto_1* | 6.9 ±6.81 | 9.98 ± 7.59 | 0.43 ± 0.82 | 9.85 ± 4.52 | 3.77E-17 | 5.53E-16 |
| *Prevotella* | 3.36 ±3.93 | 1.73 ± 1.01 | 5.05 ± 5.4 | 3.46 ± 3.55 | 4.12E-05 | 7.71E-05 |
| *Bacillus* | 1.98 ±2.46 | 2.16 ± 2.15 | 0.31 ± 0.72 | 3.37 ± 2.89 | 4.19E-13 | 3.35E-12 |
| *Bifidobacterium* | 3.57 ±8.87 | 0.01 ± 0.04 | 10.56 ± 13.34 | 0.64 ± 0.76 | 6.41E-22 | 5.64E-20 |
| *UCG-002* | 2.67 ±2.23 | 3.39 ± 2.39 | 2.07 ± 2.57 | 2.48 ± 1.41 | 1.45E-03 | 2.10E-03 |
| *Treponema* | 3.75 ±5.73 | 5.49 ± 6.65 | 2.56 ± 4.76 | 3.07 ± 5.21 | 8.79E-05 | 1.49E-04 |
| *Veillonella* | 0.95 ±4.82 | 0.23 ± 1.01 | 2.72 ± 8.27 | 0 ± 0 | 3.91E-03 | 0.01 |
| *Christensenellaceae_R-7_group* | 3.01 ±3.13 | 4.32 ± 3.86 | 2.8 ± 2.88 | 1.82 ± 1.75 | 5.22E-04 | 7.92E-04 |
| *Blautia* | 1.48 ±1.51 | 1.75 ± 1.82 | 1.29 ± 1.49 | 1.39 ± 1.1 | 0.14 | 0.15 |
| *UCG-005* | 2.29 ±1.91 | 3.08 ± 1.88 | 0.99 ± 0.98 | 2.7 ± 2.01 | 9.47E-09 | 3.21E-08 |
| *Subdoligranulum* | 1.48 ±1.31 | 1.68 ± 1.2 | 1.24 ± 1.69 | 1.51 ± 0.97 | 0.01 | 0.01 |
| *Escherichia-Shigella* | 1.64 ±2.89 | 0.71 ± 1.59 | 3.99 ± 3.89 | 0.36 ± 0.64 | 4.92E-10 | 1.97E-09 |
| *Terrisporobacter* | 2.21 ±2.53 | 1.85 ± 1.78 | 0.08 ± 0.16 | 4.62 ± 2.38 | 2.40E-19 | 7.05E-18 |
| *Enterococcus* | 1.33 ±3.08 | 0.07 ± 0.2 | 3.98 ± 4.43 | 0.12 ± 0.16 | 6.11E-18 | 1.34E-16 |
| *Rikenellaceae_RC9_gut_group* | 1.4 ±1.17 | 1.87 ± 1.04 | 0.75 ± 1.22 | 1.54 ± 0.98 | 7.08E-08 | 2.08E-07 |
| *Faecalibacterium* | 0.64 ±0.85 | 0.84 ± 1.2 | 0.39 ± 0.57 | 0.66 ± 0.52 | 4.48E-03 | 0.01 |
| *Catenibacterium* | 1.27 ±2.52 | 0.21 ± 0.34 | 3.18 ± 3.76 | 0.56 ± 0.69 | 3.82E-12 | 2.40E-11 |
| *Collinsella* | 0.75 ±1.18 | 0.48 ± 0.54 | 1.48 ± 1.8 | 0.34 ± 0.22 | 2.13E-04 | 3.53E-04 |
| *Dorea* | 0.83 ±0.72 | 0.74 ± 0.55 | 1.02 ± 1.01 | 0.75 ± 0.5 | 0.87 | 0.87 |
| *Prevotellaceae_NK3B31_group* | 1.16 ±1.9 | 0.95 ± 1.37 | 1.39 ± 2.9 | 1.16 ± 0.98 | 0.12 | 0.13 |
| *Ruminococcus* | 0.99 ±0.84 | 1.01 ± 0.71 | 0.97 ± 1.15 | 1 ± 0.6 | 0.35 | 0.36 |
| *NK4A214_group* | 1.17 ±0.92 | 1.83 ± 1.07 | 0.68 ± 0.68 | 0.95 ± 0.41 | 4.86E-09 | 1.78E-08 |
| *Dialister* | 1 ±2.21 | 0.1 ± 0.26 | 1.79 ± 3.38 | 1.19 ± 1.54 | 3.05E-08 | 9.26E-08 |
| *Phascolarctobacterium* | 0.85 ±1.07 | 0.73 ± 0.88 | 1.08 ± 1.53 | 0.75 ± 0.63 | 0.55 | 0.56 |
| *Olsenella* | 1.36 ±2.67 | 0.39 ± 0.63 | 3.24 ± 4.03 | 0.58 ± 0.84 | 1.35E-07 | 3.72E-07 |
| *Corynebacterium* | 0.47 ±0.92 | 0.01 ± 0.01 | 0.62 ± 0.89 | 0.8 ± 1.21 | 7.09E-18 | 1.25E-16 |
| *Coprococcus* | 0.71 ±0.81 | 1.36 ± 1 | 0.22 ± 0.32 | 0.49 ± 0.29 | 1.08E-15 | 1.19E-14 |
| *Megasphaera* | 0.87 ±1.6 | 0.36 ± 0.86 | 1.54 ± 2.4 | 0.75 ± 0.91 | 8.31E-08 | 2.36E-07 |
| *Holdemanella* | 0.27 ±0.33 | 0.33 ± 0.38 | 0.11 ± 0.15 | 0.35 ± 0.36 | 3.71E-05 | 7.09E-05 |
| *Lachnospiraceae_NK4A136_group* | 0.63 ±0.98 | 0.39 ± 0.34 | 1.19 ± 1.5 | 0.33 ± 0.44 | 0.05 | 0.06 |
| *Shuttleworthia* | 0.62 ±1.52 | 0.21 ± 0.7 | 0.6 ± 1.36 | 1.07 ± 2.09 | 0.01 | 0.01 |
| *Agathobacter* | 0.27 ±0.44 | 0.36 ± 0.59 | 0.17 ± 0.31 | 0.27 ± 0.32 | 0.02 | 0.03 |
| *Eubacterium_ruminantium_group* | 0.75 ±2.53 | 0.15 ± 0.14 | 1.05 ± 3.75 | 1.11 ± 2.35 | 7.12E-04 | 1.04E-03 |
| *Oribacterium* | 0.47 ±0.49 | 0.31 ± 0.3 | 0.51 ± 0.72 | 0.59 ± 0.29 | 4.13E-05 | 7.56E-05 |
| *Lachnospiraceae_XPB1014_group* | 0.78 ±1.34 | 1.18 ± 1.32 | 0.89 ± 1.76 | 0.27 ± 0.47 | 2.54E-04 | 4.06E-04 |
| *Solobacterium* | 0.45 ±2.37 | 0.9 ± 3.99 | 0.09 ± 0.11 | 0.32 ± 0.26 | 1.87E-07 | 4.85E-07 |
| *Family_XIII_AD3011_group* | 0.46 ±0.37 | 0.71 ± 0.41 | 0.27 ± 0.27 | 0.38 ± 0.22 | 1.74E-08 | 5.46E-08 |
| *Eubacterium_hallii_group* | 0.33 ±0.28 | 0.32 ± 0.29 | 0.28 ± 0.24 | 0.38 ± 0.29 | 0.06 | 0.07 |
| *Alloprevotella* | 0.36 ±0.56 | 0.71 ± 0.77 | 0.16 ± 0.2 | 0.18 ± 0.27 | 6.13E-09 | 2.16E-08 |
| *Marvinbryantia* | 0.25 ±0.21 | 0.2 ± 0.21 | 0.2 ± 0.19 | 0.33 ± 0.22 | 4.00E-04 | 6.17E-04 |
| *Ruminococcus_gauvreauii_group* | 0.29 ±0.39 | 0.39 ± 0.45 | 0.06 ± 0.12 | 0.41 ± 0.41 | 9.85E-11 | 4.82E-10 |
| *Ruminococcus_torques_group* | 0.17 ±0.35 | 0.12 ± 0.21 | 0.25 ± 0.5 | 0.16 ± 0.28 | 0.71 | 0.72 |
| *Lachnospiraceae_AC2044_group* | 0.44 ±1.05 | 0.32 ± 0.37 | 0.42 ± 1.15 | 0.58 ± 1.39 | 0.06 | 0.06 |
| *Fusobacterium* | 0.16 ±1.26 | 0.41 ± 2.12 | 0.04 ± 0.15 | 0 ± 0 | 0.06 | 0.07 |
| *Bacteroides* | 0.34 ±0.84 | 0.43 ± 0.61 | 0.31 ± 0.77 | 0.28 ± 1.09 | 0.01 | 0.01 |
| *Parabacteroides* | 0.31 ±0.38 | 0.39 ± 0.4 | 0.18 ± 0.22 | 0.35 ± 0.44 | 5.91E-04 | 8.82E-04 |
| *Lachnospira* | 0.32 ±0.52 | 0.33 ± 0.41 | 0.03 ± 0.05 | 0.58 ± 0.69 | 1.23E-10 | 5.68E-10 |
| *Roseburia* | 0.27 ±0.47 | 0.56 ± 0.67 | 0.03 ± 0.04 | 0.2 ± 0.25 | 1.70E-13 | 1.66E-12 |
| *Prevotellaceae_UCG-003* | 0.3 ±0.42 | 0.49 ± 0.58 | 0.12 ± 0.18 | 0.25 ± 0.26 | 1.33E-06 | 2.84E-06 |
| *Erysipelotrichaceae_UCG-006* | 0.25 ±0.65 | 0.15 ± 0.17 | 0.2 ± 0.6 | 0.4 ± 0.94 | 2.59E-03 | 3.62E-03 |
| *Eubacterium_eligens_group* | 0.18 ±0.25 | 0.25 ± 0.3 | 0.08 ± 0.22 | 0.2 ± 0.17 | 1.09E-06 | 2.39E-06 |
| *Prevotellaceae_UCG-001* | 0.44 ±0.93 | 0.61 ± 0.81 | 0.37 ± 1.3 | 0.32 ± 0.57 | 6.23E-06 | 1.25E-05 |
| *Sphaerochaeta* | 0.22 ±0.31 | 0.38 ± 0.38 | 0.13 ± 0.21 | 0.14 ± 0.22 | 9.33E-07 | 2.16E-06 |
| *Megamonas* | 0.13 ±0.62 | 0 ± 0.01 | 0.37 ± 1.07 | 0.03 ± 0.08 | 8.16E-05 | 1.41E-04 |
| *Fournierella* | 0.2 ±0.29 | 0.34 ± 0.42 | 0.08 ± 0.08 | 0.17 ± 0.15 | 2.72E-07 | 6.65E-07 |
| *Butyricicoccus* | 0.15 ±0.33 | 0.23 ± 0.47 | 0.05 ± 0.19 | 0.15 ± 0.21 | 4.17E-05 | 7.49E-05 |
| *Eubacterium_siraeum_group* | 0.27 ±0.39 | 0.44 ± 0.53 | 0.09 ± 0.2 | 0.28 ± 0.25 | 4.26E-10 | 1.78E-09 |
| *UCG-008* | 0.21 ±0.6 | 0.43 ± 0.92 | 0.02 ± 0.03 | 0.18 ± 0.33 | 2.53E-06 | 5.31E-06 |
| *Succinivibrio* | 0.15 ±1.16 | 0.01 ± 0.02 | 0.45 ± 2.04 | 0.02 ± 0.05 | 1.49E-07 | 3.98E-07 |
| *Turicibacter* | 0.3 ±0.4 | 0.38 ± 0.43 | 0.06 ± 0.18 | 0.46 ± 0.41 | 5.10E-12 | 2.99E-11 |
| *Acidaminococcus* | 0.16 ±0.32 | 0.01 ± 0.04 | 0.38 ± 0.47 | 0.09 ± 0.16 | 4.00E-13 | 3.52E-12 |
| *Intestinibacter* | 0.24 ±0.4 | 0.15 ± 0.18 | 0.01 ± 0.02 | 0.56 ± 0.54 | 1.15E-19 | 5.06E-18 |
| *Eubacterium_nodatum_group* | 0.24 ±0.53 | 0.25 ± 0.45 | 0.21 ± 0.6 | 0.24 ± 0.53 | 2.49E-04 | 4.06E-04 |
| *Aeriscardovia* | 0.03 ±0.31 | 0 ± 0 | 0.1 ± 0.55 | 0 ± 0 | 3.94E-03 | 0.01 |
| *Campylobacter* | 0.11 ±0.35 | 0.18 ± 0.34 | 0.01 ± 0.02 | 0.13 ± 0.48 | 1.66E-09 | 6.36E-09 |
| *Desulfovibrio* | 0.19 ±0.37 | 0.08 ± 0.12 | 0.15 ± 0.25 | 0.34 ± 0.54 | 0.03 | 0.04 |
| *Oscillospira* | 0.19 ±0.23 | 0.11 ± 0.11 | 0.02 ± 0.04 | 0.42 ± 0.25 | 4.30E-17 | 5.40E-16 |
| *Colidextribacter* | 0.1 ±0.1 | 0.09 ± 0.1 | 0.06 ± 0.07 | 0.15 ± 0.11 | 4.93E-06 | 1.01E-05 |
| *Lachnospiraceae_FCS020_group* | 0.11 ±0.11 | 0.12 ± 0.09 | 0.09 ± 0.1 | 0.13 ± 0.14 | 0.05 | 0.06 |
| *Mitsuokella* | 0.15 ±0.41 | 0.02 ± 0.07 | 0.32 ± 0.67 | 0.12 ± 0.18 | 7.89E-07 | 1.88E-06 |
| *Monoglobus* | 0.15 ±0.14 | 0.15 ± 0.11 | 0.11 ± 0.14 | 0.19 ± 0.17 | 0.01 | 0.01 |
| *Lachnoclostridium* | 0.2 ±0.32 | 0.23 ± 0.35 | 0.15 ± 0.25 | 0.22 ± 0.34 | 0.02 | 0.02 |
| *dgA-11_gut_group* | 0.15 ±0.25 | 0.28 ± 0.32 | 0.06 ± 0.12 | 0.1 ± 0.22 | 1.32E-08 | 4.29E-08 |
| *Candidatus_Soleaferrea* | 0.17 ±0.26 | 0.34 ± 0.36 | 0.06 ± 0.09 | 0.1 ± 0.08 | 1.96E-11 | 1.08E-10 |
| *Negativibacillus* | 0.14 ±0.22 | 0.09 ± 0.16 | 0.02 ± 0.05 | 0.3 ± 0.29 | 2.57E-11 | 1.33E-10 |
| *Mogibacterium* | 0.13 ±0.14 | 0.07 ± 0.07 | 0.12 ± 0.13 | 0.22 ± 0.16 | 1.93E-07 | 4.84E-07 |
| *Sutterella* | 0.03 ±0.1 | 0 ± 0.01 | 0.09 ± 0.15 | 0 ± 0.01 | 9.09E-13 | 6.67E-12 |
| *Staphylococcus* | 0.12 ±0.36 | 0.06 ± 0.1 | 0.26 ± 0.61 | 0.06 ± 0.1 | 0.01 | 0.02 |
| *Fusicatenibacter* | 0.08 ±0.15 | 0.1 ± 0.18 | 0.04 ± 0.15 | 0.09 ± 0.1 | 1.69E-05 | 3.30E-05 |
| *Lachnospiraceae_NK3A20_group* | 0.13 ±0.27 | 0.03 ± 0.05 | 0.15 ± 0.28 | 0.21 ± 0.35 | 4.50E-05 | 7.91E-05 |
| *Clostridium_sensu_stricto_6* | 0.09 ±0.21 | 0.16 ± 0.23 | 0.01 ± 0.02 | 0.09 ± 0.26 | 1.04E-12 | 7.05E-12 |
| *UCG-009* | 0.1 ±0.12 | 0.11 ± 0.12 | 0.09 ± 0.13 | 0.11 ± 0.09 | 0.03 | 0.03 |
| *Syntrophococcus* | 0.09 ±0.24 | 0.01 ± 0.04 | 0.11 ± 0.29 | 0.14 ± 0.29 | 2.57E-04 | 4.04E-04 |
| *Anaerostipes* | 0.07 ±0.2 | 0.08 ± 0.19 | 0.08 ± 0.27 | 0.05 ± 0.09 | 0.04 | 0.04 |
| *Enterorhabdus* | 0.11 ±0.15 | 0.09 ± 0.14 | 0.15 ± 0.18 | 0.09 ± 0.11 | 0.26 | 0.27 |
| *Others* | 23.92 ±8.86 | 29.59 ± 6.68 | 16.51 ± 9.28 | 25.07 ± 4.6 | 2.66E-10 | 1.17E-09 |

**Supplementary Table 4.** The compensation of microbiota for different time points of DUR stud boars at the genus level.

| **Genera** | **Relative abundance(%)** | | | | ***p*-value** | ***p*-value (corrected)** |
| --- | --- | --- | --- | --- | --- | --- |
|  | **All** | **D0** | **D7** | **D14** |  |  |
| *Lactobacillus* | 8.65 ± 12.26 | 1.93 ± 4.65 | 6.71 ± 10.24 | 15.86 ± 14.02 | 2.88E-04 | 2.30E-03 |
| *Streptococcus* | 0.48 ± 1.1 | 0.08 ± 0.09 | 1.08 ± 1.63 | 0.2 ± 0.28 | 2.18E-03 | 0.01 |
| *Clostridium_sensu_stricto_1* | 0.19 ± 0.32 | 0.19 ± 0.26 | 0.12 ± 0.21 | 0.26 ± 0.42 | 0.5 | 0.56 |
| *Prevotella* | 9.94 ± 10.41 | 18.31 ± 13.96 | 10.28 ± 6.86 | 3.01 ± 2.21 | 1.60E-04 | 1.76E-03 |
| *Bacillus* | 3.13 ± 4.03 | 0.98 ± 1.86 | 2.91 ± 4.86 | 5.05 ± 3.24 | 1.03E-03 | 0.01 |
| *Bifidobacterium* | 0.16 ± 0.52 | 0 ± 0.01 | 0.35 ± 0.8 | 0.09 ± 0.13 | 0.03 | 0.07 |
| *UCG-002* | 1.21 ± 1.73 | 0.24 ± 0.42 | 1.69 ± 2.5 | 1.5 ± 0.81 | 1.32E-03 | 0.01 |
| *Treponema* | 0.45 ± 1.1 | 0.03 ± 0.06 | 0.86 ± 1.67 | 0.38 ± 0.4 | 4.04E-03 | 0.01 |
| *Veillonella* | 0.11 ± 0.51 | 0 ± 0 | 0.02 ± 0.04 | 0.29 ± 0.8 | 2.39E-03 | 0.01 |
| *Christensenellaceae_R-7_group* | 0.73 ± 0.75 | 0.09 ± 0.12 | 0.82 ± 0.84 | 1.14 ± 0.57 | 3.46E-04 | 2.54E-03 |
| *Blautia* | 4.96 ± 6.32 | 8.41 ± 9.59 | 3.28 ± 3.21 | 3.92 ± 4.37 | 0.09 | 0.15 |
| *UCG-005* | 2.26 ± 2.87 | 3.92 ± 4.75 | 1.52 ± 1.45 | 1.7 ± 1.08 | 0.11 | 0.16 |
| *Subdoligranulum* | 2 ± 1.99 | 3.94 ± 2.44 | 0.67 ± 0.83 | 1.8 ± 1.04 | 4.43E-05 | 1.95E-03 |
| *Escherichia-Shigella* | 2.58 ± 4.38 | 0.54 ± 0.54 | 6.38 ± 5.39 | 0.39 ± 0.52 | 6.45E-05 | 1.42E-03 |
| *Terrisporobacter* | 0.23 ± 0.65 | 0.71 ± 1.12 | 0.03 ± 0.07 | 0.04 ± 0.08 | 4.11E-03 | 0.01 |
| *Enterococcus* | 2.09 ± 6.47 | 0 ± 0 | 5.72 ± 9.64 | 0.09 ± 0.15 | 4.70E-06 | 4.14E-04 |
| *Rikenellaceae_RC9_gut_group* | 2.03 ± 2.17 | 1.45 ± 2.32 | 1.63 ± 1.29 | 2.87 ± 2.47 | 0.04 | 0.09 |
| *Faecalibacterium* | 2.1 ± 2.84 | 4.69 ± 3.74 | 0.62 ± 0.58 | 1.55 ± 1.93 | 1.07E-03 | 0.01 |
| *Catenibacterium* | 2.63 ± 4.9 | 1.52 ± 1.59 | 5.4 ± 7.09 | 0.74 ± 0.95 | 1.36E-03 | 0.01 |
| *Collinsella* | 1.92 ± 2.28 | 1.18 ± 1.89 | 2.26 ± 1.97 | 2.16 ± 2.65 | 0.11 | 0.17 |
| *Dorea* | 1.58 ± 1.49 | 0.86 ± 0.53 | 2.3 ± 2.01 | 1.43 ± 0.91 | 0.11 | 0.16 |
| *Prevotellaceae_NK3B31_group* | 2.47 ± 3.51 | 3.65 ± 3.9 | 2.75 ± 4.31 | 1.26 ± 1.16 | 0.19 | 0.26 |
| *Ruminococcus* | 1.16 ± 1.14 | 1.28 ± 1.51 | 0.83 ± 0.95 | 1.38 ± 0.92 | 0.1 | 0.16 |
| *NK4A214_group* | 1.04 ± 1.53 | 0.39 ± 0.59 | 1.31 ± 2.16 | 1.28 ± 1 | 0.01 | 0.03 |
| *Dialister* | 0.03 ± 0.13 | 0 ± 0 | 0.01 ± 0.04 | 0.06 ± 0.2 | 0.04 | 0.08 |
| *Phascolarctobacterium* | 2.55 ± 2.56 | 4.69 ± 2.26 | 1.9 ± 2.37 | 1.51 ± 1.85 | 3.76E-03 | 0.01 |
| *Olsenella* | 0.43 ± 0.55 | 0.66 ± 0.76 | 0.34 ± 0.28 | 0.35 ± 0.54 | 0.43 | 0.5 |
| *Corynebacterium* | 0.35 ± 0.59 | 0.04 ± 0.04 | 0.34 ± 0.74 | 0.6 ± 0.5 | 3.27E-03 | 0.01 |
| *Coprococcus* | 0.99 ± 0.96 | 1.27 ± 1.11 | 1.14 ± 1.11 | 0.63 ± 0.45 | 0.41 | 0.48 |
| *Megasphaera* | 0.75 ± 1.9 | 0.55 ± 0.92 | 0.29 ± 0.55 | 1.37 ± 2.88 | 0.44 | 0.51 |
| *Holdemanella* | 0.79 ± 0.86 | 1.18 ± 1.22 | 0.46 ± 0.53 | 0.8 ± 0.67 | 0.08 | 0.14 |
| *Lachnospiraceae_NK4A136_group* | 0.44 ± 0.56 | 0.4 ± 0.59 | 0.24 ± 0.21 | 0.67 ± 0.68 | 0.1 | 0.16 |
| *Shuttleworthia* | 0 ± 0 | 0 ± 0 | 0 ± 0 | 0 ± 0 | 0.07 | 0.13 |
| *Agathobacter* | 0.15 ± 0.37 | 0.02 ± 0.04 | 0.06 ± 0.1 | 0.35 ± 0.54 | 0.03 | 0.06 |
| *Eubacterium_ruminantium_group* | 0.03 ± 0.07 | 0 ± 0 | 0.01 ± 0.02 | 0.07 ± 0.1 | 1.09E-03 | 0.01 |
| *Oribacterium* | 0.27 ± 0.33 | 0.19 ± 0.37 | 0.29 ± 0.34 | 0.31 ± 0.28 | 0.11 | 0.16 |
| *Lachnospiraceae_XPB1014_group* | 0.01 ± 0.06 | 0 ± 0 | 0 ± 0.01 | 0.03 ± 0.09 | 0.4 | 0.48 |
| *Solobacterium* | 0.26 ± 0.29 | 0.24 ± 0.39 | 0.21 ± 0.26 | 0.32 ± 0.22 | 0.13 | 0.18 |
| *Family_XIII_AD3011_group* | 1.28 ± 2.75 | 2.73 ± 4.8 | 0.51 ± 0.53 | 0.91 ± 1.2 | 0.26 | 0.34 |
| *Eubacterium_hallii_group* | 0.81 ± 0.67 | 0.97 ± 0.71 | 0.79 ± 0.74 | 0.7 ± 0.54 | 0.64 | 0.69 |
| *Alloprevotella* | 0.7 ± 0.82 | 0.96 ± 0.8 | 0.94 ± 1.03 | 0.26 ± 0.14 | 0.01 | 0.02 |
| *Marvinbryantia* | 0.39 ± 0.37 | 0.25 ± 0.19 | 0.25 ± 0.18 | 0.64 ± 0.47 | 0.01 | 0.02 |
| *Ruminococcus_gauvreauii_group* | 0.25 ± 0.37 | 0.14 ± 0.15 | 0.21 ± 0.48 | 0.38 ± 0.33 | 0.07 | 0.13 |
| *Ruminococcus_torques_group* | 0.42 ± 0.69 | 0.01 ± 0.03 | 0.54 ± 0.94 | 0.61 ± 0.46 | 5.75E-05 | 1.69E-03 |
| *Lachnospiraceae_AC2044_group* | 0.68 ± 2.08 | 0.04 ± 0.09 | 0.1 ± 0.25 | 1.76 ± 3.14 | 0.04 | 0.09 |
| *Fusobacterium* | 0.69 ± 3.91 | 0.01 ± 0.02 | 1.92 ± 6.25 | 0 ± 0 | 0.01 | 0.03 |
| *Bacteroides* | 0.35 ± 0.5 | 0.23 ± 0.22 | 0.5 ± 0.72 | 0.29 ± 0.31 | 0.64 | 0.69 |
| *Parabacteroides* | 0.71 ± 1.4 | 1.4 ± 2.51 | 0.41 ± 0.36 | 0.47 ± 0.42 | 0.87 | 0.91 |
| *Lachnospira* | 0.03 ± 0.06 | 0.02 ± 0.06 | 0.02 ± 0.03 | 0.05 ± 0.08 | 0.29 | 0.37 |
| *Roseburia* | 0.38 ± 0.62 | 0.65 ± 1.05 | 0.08 ± 0.12 | 0.45 ± 0.28 | 8.12E-04 | 0.01 |
| *Prevotellaceae_UCG-003* | 0.32 ± 0.33 | 0.16 ± 0.12 | 0.43 ± 0.39 | 0.34 ± 0.31 | 0.08 | 0.14 |
| *Erysipelotrichaceae_UCG-006* | 0.05 ± 0.06 | 0.04 ± 0.06 | 0.03 ± 0.04 | 0.09 ± 0.07 | 0.02 | 0.06 |
| *Eubacterium_eligens_group* | 0.11 ± 0.13 | 0.12 ± 0.14 | 0.05 ± 0.07 | 0.16 ± 0.14 | 0.02 | 0.05 |
| *Prevotellaceae_UCG-001* | 0.11 ± 0.28 | 0.25 ± 0.47 | 0.09 ± 0.18 | 0.03 ± 0.05 | 0.09 | 0.15 |
| *Sphaerochaeta* | 0.22 ± 0.34 | 0.07 ± 0.13 | 0.41 ± 0.46 | 0.14 ± 0.13 | 4.69E-03 | 0.02 |
| *Megamonas* | 0.02 ± 0.04 | 0 ± 0 | 0.02 ± 0.03 | 0.04 ± 0.06 | 0.05 | 0.09 |
| *Fournierella* | 0.23 ± 0.19 | 0.24 ± 0.15 | 0.17 ± 0.14 | 0.27 ± 0.23 | 0.47 | 0.53 |
| *Butyricicoccus* | 0.27 ± 0.44 | 0.33 ± 0.38 | 0.27 ± 0.39 | 0.24 ± 0.52 | 0.3 | 0.37 |
| *Eubacterium_siraeum_group* | 0.18 ± 0.27 | 0.13 ± 0.2 | 0.09 ± 0.14 | 0.3 ± 0.35 | 0.02 | 0.06 |
| *UCG-008* | 0.59 ± 1.4 | 0.81 ± 1.36 | 0.03 ± 0.05 | 0.97 ± 1.88 | 0.09 | 0.15 |
| *Succinivibrio* | 0.02 ± 0.07 | 0 ± 0.01 | 0.05 ± 0.11 | 0 ± 0.01 | 0.01 | 0.02 |
| *Turicibacter* | 0 ± 0 | 0 ± 0 | 0 ± 0 | 0 ± 0 | 1 | 1.01 |
| *Acidaminococcus* | 0.16 ± 0.4 | 0.23 ± 0.54 | 0.15 ± 0.45 | 0.11 ± 0.15 | 0.71 | 0.75 |
| *Intestinibacter* | 0.01 ± 0.03 | 0 ± 0.02 | 0 ± 0 | 0.01 ± 0.05 | 0.98 | 1.01 |
| *Eubacterium_nodatum_group* | 0.04 ± 0.06 | 0.03 ± 0.05 | 0.03 ± 0.04 | 0.07 ± 0.08 | 0.26 | 0.34 |
| *Aeriscardovia* | 0 ± 0 | 0 ± 0 | 0 ± 0 | 0 ± 0 | 1 | 1 |
| *Campylobacter* | 0.05 ± 0.11 | 0.03 ± 0.06 | 0.03 ± 0.03 | 0.09 ± 0.16 | 0.25 | 0.34 |
| *Desulfovibrio* | 0.02 ± 0.04 | 0 ± 0 | 0.02 ± 0.02 | 0.03 ± 0.06 | 2.93E-03 | 0.01 |
| *Oscillospira* | 0.2 ± 0.29 | 0.57 ± 0.33 | 0.06 ± 0.06 | 0.06 ± 0.08 | 1.17E-04 | 1.47E-03 |
| *Colidextribacter* | 0.53 ± 0.54 | 0.77 ± 0.53 | 0.73 ± 0.6 | 0.14 ± 0.08 | 1.04E-04 | 1.83E-03 |
| *Lachnospiraceae_FCS020_group* | 0.16 ± 0.17 | 0.1 ± 0.1 | 0.17 ± 0.21 | 0.2 ± 0.15 | 0.09 | 0.15 |
| *Mitsuokella* | 0.16 ± 0.37 | 0.23 ± 0.34 | 0.23 ± 0.51 | 0.04 ± 0.11 | 0.51 | 0.56 |
| *Monoglobus* | 0.11 ± 0.21 | 0 ± 0.01 | 0.13 ± 0.3 | 0.16 ± 0.12 | 1.09E-04 | 1.59E-03 |
| *Lachnoclostridium* | 0.06 ± 0.13 | 0.03 ± 0.04 | 0.04 ± 0.06 | 0.11 ± 0.18 | 0.03 | 0.07 |
| *dgA-11_gut_group* | 0.07 ± 0.14 | 0.01 ± 0.03 | 0.07 ± 0.16 | 0.12 ± 0.14 | 2.22E-03 | 0.01 |
| *Candidatus_Soleaferrea* | 0.19 ± 0.46 | 0.16 ± 0.3 | 0.12 ± 0.19 | 0.28 ± 0.67 | 0.38 | 0.46 |
| *Negativibacillus* | 0.25 ± 0.62 | 0.34 ± 0.23 | 0.3 ± 0.99 | 0.13 ± 0.14 | 2.25E-04 | 1.98E-03 |
| *Mogibacterium* | 0.21 ± 0.81 | 0.07 ± 0.08 | 0.07 ± 0.09 | 0.46 ± 1.29 | 0.05 | 0.09 |
| *Sutterella* | 0.03 ± 0.12 | 0 ± 0 | 0.09 ± 0.19 | 0 ± 0.01 | 1.66E-04 | 1.63E-03 |
| *Staphylococcus* | 0.07 ± 0.2 | 0 ± 0 | 0.17 ± 0.3 | 0.02 ± 0.04 | 0.04 | 0.09 |
| *Fusicatenibacter* | 0.11 ± 0.17 | 0.01 ± 0.02 | 0.1 ± 0.1 | 0.2 ± 0.23 | 0.01 | 0.03 |
| *Lachnospiraceae_NK3A20_group* | 0.01 ± 0.04 | 0 ± 0 | 0.02 ± 0.06 | 0.01 ± 0.01 | 0.1 | 0.16 |
| *Clostridium_sensu_stricto_6* | 0.16 ± 0.31 | 0.03 ± 0.05 | 0.2 ± 0.32 | 0.21 ± 0.37 | 0.21 | 0.29 |
| *UCG-009* | 0.24 ± 0.33 | 0.1 ± 0.18 | 0.26 ± 0.36 | 0.32 ± 0.34 | 0.02 | 0.06 |
| *Syntrophococcus* | 0.04 ± 0.13 | 0.04 ± 0.1 | 0.03 ± 0.08 | 0.06 ± 0.19 | 0.98 | 1.01 |
| *Anaerostipes* | 0.13 ± 0.21 | 0.26 ± 0.33 | 0.06 ± 0.09 | 0.09 ± 0.09 | 0.32 | 0.39 |
| *Enterorhabdus* | 0.13 ± 0.12 | 0.12 ± 0.12 | 0.1 ± 0.08 | 0.17 ± 0.13 | 0.47 | 0.53 |
| *Others* | 26.91 ± 9.86 | 23.63 ± 8.89 | 24.09 ± 9.83 | 32.3 ± 8.1 | 0.06 | 0.11 |

**Supplementary Table 5.** Different enriched KOs and KEGG pathways among different time points of LW stud boar samples.

| **Item** | **LDA Score** | ***p*-value (corrected)** | **Group** |
| --- | --- | --- | --- |
| ko02020:Signal transduction | 3.01 | 1.55E-11 | D0 |
| ko01212:Global and overview maps | 2.64 | 2.72E-11 | D0 |
| ko00190:Energy metabolism | 2.62 | 5.07E-11 | D0 |
| ko00250:Amino acid metabolism | 2.51 | 4.71E-12 | D0 |
| ko01120:Global and overview maps | 2.48 | 1.28E-02 | D0 |
| ko01200:Global and overview maps | 2.45 | 4.99E-03 | D0 |
| ko00620:Carbohydrate metabolism | 2.45 | 2.06E-04 | D0 |
| ko00061:Lipid metabolism | 2.35 | 7.28E-05 | D0 |
| ko00220:Amino acid metabolism | 2.32 | 6.67E-05 | D0 |
| ko01502:Drug resistance: antimicrobial | 2.28 | 3.84E-13 | D0 |
| ko00460:Metabolism of other amino acids | 2.27 | 2.01E-11 | D0 |
| ko04122:Folding, sorting and degradation | 2.25 | 1.17E-09 | D0 |
| ko00511:Glycan biosynthesis and metabolism | 2.22 | 4.90E-07 | D0 |
| ko00410:Metabolism of other amino acids | 2.21 | 3.55E-11 | D0 |
| ko04141:Folding, sorting and degradation | 2.2 | 4.05E-12 | D0 |
| ko00650:Carbohydrate metabolism | 2.16 | 3.81E-03 | D0 |
| ko00240:Nucleotide metabolism | 2.13 | 3.70E-02 | D0 |
| ko04727:Nervous system | 2.1 | 1.65E-12 | D0 |
| ko00310:Amino acid metabolism | 2.07 | 1.21E-04 | D0 |
| ko00640:Carbohydrate metabolism | 2.07 | 2.01E-02 | D0 |
| ko00940:Biosynthesis of other secondary metabolites | 2.05 | 5.85E-05 | D0 |
| ko00983:Xenobiotics biodegradation and metabolism | 2.01 | 2.90E-06 | D0 |
| ko05200:Cancer: overview | 2.01 | 1.51E-13 | D0 |
| ko04112:Cell growth and death | 1.99 | 2.06E-04 | D0 |
| ko00380:Amino acid metabolism | 1.98 | 3.13E-06 | D0 |
| ko00360:Amino acid metabolism | 1.98 | 1.80E-02 | D0 |
| ko00523:Metabolism of terpenoids and polyketides | 1.9 | 2.60E-03 | D0 |
| ko00900:Metabolism of terpenoids and polyketides | 1.9 | 1.77E-02 | D0 |
| ko00261:Biosynthesis of other secondary metabolites | 1.9 | 2.74E-02 | D0 |
| ko00998:Biosynthesis of other secondary metabolites | 1.87 | 3.06E-08 | D0 |
| ko05206:Cancer: overview | 1.87 | 1.16E-09 | D0 |
| ko00670:Metabolism of cofactors and vitamins | 1.85 | 3.73E-02 | D0 |
| ko00071:Lipid metabolism | 1.78 | 1.37E-03 | D0 |
| ko00600:Lipid metabolism | 1.76 | 3.76E-02 | D0 |
| ko00440:Metabolism of other amino acids | 1.73 | 2.04E-05 | D0 |
| ko03320:Endocrine system | 1.73 | 1.10E-02 | D0 |
| ko00525:Biosynthesis of other secondary metabolites | 1.61 | 6.16E-03 | D0 |
| ko00072:Lipid metabolism | 1.61 | 1.12E-02 | D0 |
| ko04361:Development and regeneration | 1.59 | 1.23E-09 | D0 |
| ko04072:Signal transduction | 1.59 | 1.23E-09 | D0 |
| ko04138:Transport and catabolism | 1.58 | 6.65E-09 | D0 |
| ko05133:Infectious disease: bacterial | 1.58 | 3.71E-03 | D0 |
| ko00604:Glycan biosynthesis and metabolism | 1.45 | 3.76E-05 | D0 |
| ko00513:Glycan biosynthesis and metabolism | 1.45 | 4.96E-05 | D0 |
| ko00643:Xenobiotics biodegradation and metabolism | 1.41 | 5.32E-05 | D0 |
| ko01054:Metabolism of terpenoids and polyketides | 1.39 | 4.08E-02 | D0 |
| ko01055:Metabolism of terpenoids and polyketides | 1.35 | 6.97E-03 | D0 |
| ko04210:Cell growth and death | 1.35 | 8.12E-04 | D0 |
| ko05211:Cancer: specific types | 1.32 | 3.98E-02 | D0 |
| ko04934:Endocrine and metabolic disease | 1.32 | 3.98E-02 | D0 |
| ko04940:Endocrine and metabolic disease | 1.3 | 6.27E-04 | D0 |
| ko01523:Drug resistance: antineoplastic | 1.3 | 4.08E-02 | D0 |
| ko00785:Metabolism of cofactors and vitamins | 1.29 | 4.43E-02 | D0 |
| ko00592:Lipid metabolism | 1.21 | 8.88E-06 | D0 |
| ko00281:Metabolism of terpenoids and polyketides | 1.19 | 3.35E-02 | D0 |
| ko05145:Infectious disease: parasitic | 1.16 | 1.23E-09 | D0 |
| ko04932:Endocrine and metabolic disease | 1.08 | 1.54E-15 | D0 |
| ko05012:Neurodegenerative disease | 1.08 | 1.60E-15 | D0 |
| ko00642:Xenobiotics biodegradation and metabolism | 1.06 | 4.25E-06 | D0 |
| ko00591:Lipid metabolism | 0.95 | 1.17E-05 | D0 |
| ko00623:Xenobiotics biodegradation and metabolism | 0.92 | 2.42E-02 | D0 |
| ko00565:Lipid metabolism | 0.9 | 8.77E-05 | D0 |
| ko05130:Infectious disease: bacterial | 0.87 | 4.89E-02 | D0 |
| ko03022:Transcription | 0.84 | 2.49E-03 | D0 |
| ko04113:Cell growth and death | 0.81 | 1.58E-02 | D0 |
| ko05161:Infectious disease: viral | 0.79 | 1.54E-15 | D0 |
| ko05160:Infectious disease: viral | 0.79 | 1.54E-15 | D0 |
| ko05168:Infectious disease: viral | 0.79 | 1.54E-15 | D0 |
| ko05162:Infectious disease: viral | 0.79 | 1.54E-15 | D0 |
| ko05164:Infectious disease: viral | 0.79 | 1.54E-15 | D0 |
| ko04215:Cell growth and death | 0.79 | 1.54E-15 | D0 |
| ko04115:Cell growth and death | 0.79 | 1.54E-15 | D0 |
| ko05210:Cancer: specific types | 0.79 | 1.54E-15 | D0 |
| ko05416:Cardiovascular disease | 0.79 | 1.54E-15 | D0 |
| ko05169:Infectious disease: viral | 0.79 | 1.54E-15 | D0 |
| ko05222:Cancer: specific types | 0.79 | 1.54E-15 | D0 |
| ko05170:Infectious disease: viral | 0.79 | 1.54E-15 | D0 |
| ko05163:Infectious disease: viral | 0.79 | 1.54E-15 | D0 |
| ko05167:Infectious disease: viral | 0.79 | 1.54E-15 | D0 |
| ko00140:Lipid metabolism | 0.78 | 1.28E-03 | D0 |
| ko04260:Circulatory system | 0.72 | 1.04E-08 | D0 |
| ko04723:Nervous system | 0.42 | 1.09E-13 | D0 |
| ko05110:Infectious disease: bacterial | 0.34 | 6.22E-10 | D0 |
| ko00941:Biosynthesis of other secondary metabolites | 0.28 | 4.51E-02 | D0 |
| ko00945:Biosynthesis of other secondary metabolites | 0.28 | 4.51E-02 | D0 |
| ko05017:Neurodegenerative disease | 0.16 | 1.46E-03 | D0 |
| ko00196:Energy metabolism | 0.06 | 3.10E-03 | D0 |
| ko03040:Transcription | 0.05 | 8.82E-03 | D0 |
| ko04640:Immune system | 0.05 | 2.72E-02 | D0 |
| ko00515:Glycan biosynthesis and metabolism | 0.04 | 3.48E-02 | D0 |
| ko00514:Glycan biosynthesis and metabolism | 0.04 | 3.48E-02 | D0 |
| ko01057:Metabolism of terpenoids and polyketides | 0.03 | 8.86E-04 | D0 |
| ko04110:Cell growth and death | 0.03 | 1.90E-02 | D0 |
| ko04916:Endocrine system | 0.03 | 5.94E-03 | D0 |
| ko04921:Endocrine system | 0.03 | 2.60E-03 | D0 |
| ko04371:Signal transduction | 0.03 | 2.26E-03 | D0 |
| ko00232:Biosynthesis of other secondary metabolites | 0.02 | 8.60E-04 | D0 |
| ko04020:Signal transduction | 0.02 | 3.44E-04 | D0 |
| ko04261:Circulatory system | 0.02 | 3.44E-04 | D0 |
| ko04713:Environmental adaptation | 0.02 | 3.44E-04 | D0 |
| ko04924:Endocrine system | 0.02 | 3.80E-03 | D0 |
| ko04625:Immune system | 0.02 | 9.17E-04 | D0 |
| ko05166:Infectious disease: viral | 0.02 | 2.95E-02 | D0 |
| ko04014:Signal transduction | 0.02 | 3.35E-03 | D0 |
| ko04912:Endocrine system | 0.02 | 3.35E-03 | D0 |
| ko04218:Cell growth and death | 0.02 | 8.86E-04 | D0 |
| ko04114:Cell growth and death | 0.02 | 8.86E-04 | D0 |
| ko04015:Signal transduction | 0.02 | 8.86E-04 | D0 |
| ko04022:Signal transduction | 0.02 | 8.86E-04 | D0 |
| ko05214:Cancer: specific types | 0.02 | 8.86E-04 | D0 |
| ko04270:Circulatory system | 0.02 | 8.86E-04 | D0 |
| ko04971:Digestive system | 0.02 | 8.86E-04 | D0 |
| ko04970:Digestive system | 0.02 | 8.86E-04 | D0 |
| ko04925:Endocrine system | 0.02 | 8.86E-04 | D0 |
| ko04720:Nervous system | 0.02 | 8.86E-04 | D0 |
| ko04722:Nervous system | 0.02 | 8.86E-04 | D0 |
| ko04750:Sensory system | 0.02 | 8.86E-04 | D0 |
| ko04740:Sensory system | 0.02 | 8.86E-04 | D0 |
| ko04745:Sensory system | 0.02 | 8.86E-04 | D0 |
| ko04744:Sensory system | 0.02 | 8.86E-04 | D0 |
| ko03015:Translation | 0.01 | 2.95E-02 | D0 |
| ko04140:Transport and catabolism | 0.01 | 2.95E-02 | D0 |
| ko04145:Transport and catabolism | 0.01 | 2.95E-02 | D0 |
| ko04150:Signal transduction | 0.01 | 2.95E-02 | D0 |
| ko01521:Drug resistance: antineoplastic | 0.01 | 2.95E-02 | D0 |
| ko03460:Replication and repair | 0.01 | 2.95E-02 | D0 |
| ko02040:Cell motility | 3.28 | 7.00E-14 | D14 |
| ko02030:Cell motility | 3.17 | 6.63E-14 | D14 |
| ko02026:Cellular community - prokaryotes | 2.5 | 5.24E-07 | D14 |
| ko04626:Environmental adaptation | 2.47 | 8.18E-14 | D14 |
| ko04621:Immune system | 2.47 | 4.59E-14 | D14 |
| ko05132:Infectious disease: bacterial | 2.45 | 5.80E-14 | D14 |
| ko04152:Signal transduction | 2.34 | 3.84E-13 | D14 |
| ko03018:Folding, sorting and degradation | 2.34 | 1.78E-08 | D14 |
| ko00333:Biosynthesis of other secondary metabolites | 2.29 | 4.87E-14 | D14 |
| ko05418:Cardiovascular disease | 2.21 | 8.71E-13 | D14 |
| ko04217:Cell growth and death | 2.17 | 5.02E-06 | D14 |
| ko04151:Signal transduction | 2.15 | 2.21E-12 | D14 |
| ko05111:Cellular community - prokaryotes | 2.11 | 3.44E-04 | D14 |
| ko00521:Biosynthesis of other secondary metabolites | 2.11 | 4.04E-03 | D14 |
| ko01051:Metabolism of terpenoids and polyketides | 2.09 | 1.02E-11 | D14 |
| ko00730:Metabolism of cofactors and vitamins | 2.07 | 6.49E-04 | D14 |
| ko04910:Endocrine system | 2.07 | 2.86E-06 | D14 |
| ko00564:Lipid metabolism | 2.05 | 2.26E-03 | D14 |
| ko04724:Nervous system | 2.04 | 1.06E-12 | D14 |
| ko04915:Endocrine system | 2.01 | 6.22E-12 | D14 |
| ko05215:Cancer: specific types | 2.01 | 6.22E-12 | D14 |
| ko04914:Endocrine system | 2.01 | 6.22E-12 | D14 |
| ko04612:Immune system | 2.01 | 6.22E-12 | D14 |
| ko04659:Immune system | 2.01 | 6.22E-12 | D14 |
| ko04657:Immune system | 2.01 | 6.22E-12 | D14 |
| ko05131:Infectious disease: bacterial | 1.99 | 7.03E-09 | D14 |
| ko03013:Translation | 1.97 | 9.86E-11 | D14 |
| ko04931:Endocrine and metabolic disease | 1.94 | 7.34E-05 | D14 |
| ko02025:Cellular community - prokaryotes | 1.94 | 8.86E-04 | D14 |
| ko00524:Biosynthesis of other secondary metabolites | 1.93 | 2.38E-15 | D14 |
| ko05120:Infectious disease: bacterial | 1.89 | 4.66E-05 | D14 |
| ko05134:Infectious disease: bacterial | 1.84 | 1.33E-07 | D14 |
| ko00633:Xenobiotics biodegradation and metabolism | 1.82 | 1.10E-03 | D14 |
| ko00966:Biosynthesis of other secondary metabolites | 1.81 | 8.11E-04 | D14 |
| ko03430:Replication and repair | 1.76 | 4.37E-02 | D14 |
| ko00981:Metabolism of terpenoids and polyketides | 1.64 | 8.09E-09 | D14 |
| ko00121:Lipid metabolism | 1.64 | 5.22E-04 | D14 |
| ko00903:Metabolism of terpenoids and polyketides | 1.6 | 6.06E-07 | D14 |
| ko05231:Cancer: overview | 1.58 | 5.99E-09 | D14 |
| ko04213:Aging | 1.54 | 2.29E-02 | D14 |
| ko05165:Infectious disease: viral | 1.35 | 4.51E-07 | D14 |
| ko05203:Cancer: overview | 1.35 | 4.54E-07 | D14 |
| ko04930:Endocrine and metabolic disease | 1.28 | 3.03E-05 | D14 |
| ko04978:Digestive system | 1.18 | 1.09E-09 | D14 |
| ko03450:Replication and repair | 1.14 | 1.25E-12 | D14 |
| ko00791:Xenobiotics biodegradation and metabolism | 1.11 | 7.01E-04 | D14 |
| ko05020:Neurodegenerative disease | 0.92 | 5.66E-04 | D14 |
| ko00906:Metabolism of terpenoids and polyketides | 0.87 | 9.68E-04 | D14 |
| ko04614:Endocrine system | 0.71 | 2.42E-02 | D14 |
| ko00984:Xenobiotics biodegradation and metabolism | 0.63 | 5.07E-11 | D14 |
| ko04919:Endocrine system | 0.6 | 3.61E-12 | D14 |
| ko04024:Signal transduction | 0.42 | 1.78E-11 | D14 |
| ko04071:Signal transduction | 0.38 | 8.59E-11 | D14 |
| ko05034:Substance dependence | 0.34 | 8.18E-14 | D14 |
| ko05031:Substance dependence | 0.34 | 8.18E-14 | D14 |
| ko04728:Nervous system | 0.34 | 8.18E-14 | D14 |
| ko05030:Substance dependence | 0.33 | 8.18E-14 | D14 |
| ko04726:Nervous system | 0.33 | 8.18E-14 | D14 |
| ko00997:Biosynthesis of other secondary metabolites | 0.27 | 2.15E-12 | D14 |
| ko04976:Digestive system | 0.11 | 7.92E-04 | D14 |
| ko04928:Endocrine system | 0.04 | 1.76E-03 | D14 |
| ko04962:Excretory system | 0.03 | 3.24E-04 | D14 |
| ko04721:Nervous system | 0.03 | 3.24E-04 | D14 |
| ko01100:Global and overview maps | 3.41 | 6.22E-12 | D7 |
| ko02060:Membrane transport | 3.2 | 3.30E-10 | D7 |
| ko02010:Membrane transport | 3.16 | 1.19E-06 | D7 |
| ko00540:Glycan biosynthesis and metabolism | 3.01 | 6.22E-09 | D7 |
| ko00500:Carbohydrate metabolism | 2.92 | 9.65E-04 | D7 |
| ko02024:Cellular community - prokaryotes | 2.72 | 7.83E-05 | D7 |
| ko00790:Metabolism of cofactors and vitamins | 2.69 | 3.85E-09 | D7 |
| ko01501:Drug resistance: antimicrobial | 2.68 | 6.20E-10 | D7 |
| ko00520:Carbohydrate metabolism | 2.67 | 1.57E-04 | D7 |
| ko00051:Carbohydrate metabolism | 2.65 | 5.91E-08 | D7 |
| ko00130:Metabolism of cofactors and vitamins | 2.63 | 4.26E-11 | D7 |
| ko00480:Metabolism of other amino acids | 2.56 | 3.17E-10 | D7 |
| ko00052:Carbohydrate metabolism | 2.54 | 1.24E-02 | D7 |
| ko00010:Carbohydrate metabolism | 2.54 | 3.27E-02 | D7 |
| ko00020:Carbohydrate metabolism | 2.32 | 2.13E-02 | D7 |
| ko01503:Drug resistance: antimicrobial | 2.3 | 3.59E-06 | D7 |
| ko00450:Metabolism of other amino acids | 2.25 | 1.25E-12 | D7 |
| ko01220:Global and overview maps | 2.24 | 1.49E-05 | D7 |
| ko00260:Amino acid metabolism | 2.24 | 6.67E-05 | D7 |
| ko05150:Infectious disease: bacterial | 2.21 | 3.40E-04 | D7 |
| ko00053:Carbohydrate metabolism | 2.16 | 1.94E-05 | D7 |
| ko00473:Metabolism of other amino acids | 2.09 | 4.25E-06 | D7 |
| ko00311:Biosynthesis of other secondary metabolites | 2.09 | 6.22E-12 | D7 |
| ko04146:Transport and catabolism | 2.08 | 1.65E-05 | D7 |
| ko04973:Digestive system | 2.08 | 1.06E-12 | D7 |
| ko00270:Amino acid metabolism | 2.08 | 4.00E-03 | D7 |
| ko04972:Digestive system | 2.07 | 8.27E-13 | D7 |
| ko00572:Glycan biosynthesis and metabolism | 2.03 | 8.18E-14 | D7 |
| ko00362:Xenobiotics biodegradation and metabolism | 2.02 | 2.72E-04 | D7 |
| ko04016:Signal transduction | 1.95 | 1.78E-11 | D7 |
| ko04714:Environmental adaptation | 1.94 | 1.27E-06 | D7 |
| ko00740:Metabolism of cofactors and vitamins | 1.93 | 9.68E-04 | D7 |
| ko00750:Metabolism of cofactors and vitamins | 1.93 | 2.99E-03 | D7 |
| ko03020:Transcription | 1.89 | 1.79E-03 | D7 |
| ko04216:Cell growth and death | 1.89 | 6.14E-05 | D7 |
| ko01053:Metabolism of terpenoids and polyketides | 1.89 | 3.11E-12 | D7 |
| ko00621:Xenobiotics biodegradation and metabolism | 1.85 | 7.63E-07 | D7 |
| ko00622:Xenobiotics biodegradation and metabolism | 1.84 | 1.31E-05 | D7 |
| ko04917:Endocrine system | 1.83 | 2.00E-07 | D7 |
| ko00350:Amino acid metabolism | 1.81 | 2.96E-03 | D7 |
| ko00120:Lipid metabolism | 1.79 | 6.62E-04 | D7 |
| ko00405:Biosynthesis of other secondary metabolites | 1.78 | 9.38E-06 | D7 |
| ko04918:Endocrine system | 1.76 | 4.51E-07 | D7 |
| ko00571:Glycan biosynthesis and metabolism | 1.74 | 1.54E-15 | D7 |
| ko04920:Endocrine system | 1.73 | 6.71E-03 | D7 |
| ko05146:Infectious disease: parasitic | 1.72 | 2.96E-05 | D7 |
| ko00627:Xenobiotics biodegradation and metabolism | 1.7 | 1.42E-02 | D7 |
| ko05143:Infectious disease: parasitic | 1.66 | 1.44E-10 | D7 |
| ko05340:Immune disease | 1.66 | 1.94E-02 | D7 |
| ko00472:Metabolism of other amino acids | 1.65 | 1.83E-09 | D7 |
| ko01524:Drug resistance: antineoplastic | 1.64 | 2.58E-07 | D7 |
| ko04212:Aging | 1.63 | 2.47E-04 | D7 |
| ko04211:Aging | 1.63 | 2.68E-06 | D7 |
| ko04013:Signal transduction | 1.63 | 1.39E-06 | D7 |
| ko00361:Xenobiotics biodegradation and metabolism | 1.62 | 5.94E-07 | D7 |
| ko05152:Infectious disease: bacterial | 1.62 | 3.68E-06 | D7 |
| ko05016:Neurodegenerative disease | 1.61 | 1.07E-02 | D7 |
| ko00980:Xenobiotics biodegradation and metabolism | 1.58 | 1.75E-02 | D7 |
| ko00965:Biosynthesis of other secondary metabolites | 1.57 | 2.03E-08 | D7 |
| ko00982:Xenobiotics biodegradation and metabolism | 1.57 | 2.28E-02 | D7 |
| ko00944:Biosynthesis of other secondary metabolites | 1.56 | 8.51E-09 | D7 |
| ko01040:Lipid metabolism | 1.53 | 8.07E-12 | D7 |
| ko05100:Infectious disease: bacterial | 1.51 | 9.19E-08 | D7 |
| ko03008:Translation | 1.48 | 3.23E-07 | D7 |
| ko04214:Cell growth and death | 1.46 | 1.86E-05 | D7 |
| ko00510:Glycan biosynthesis and metabolism | 1.42 | 5.10E-04 | D7 |
| ko03050:Folding, sorting and degradation | 1.41 | 4.24E-17 | D7 |
| ko05142:Infectious disease: parasitic | 1.36 | 4.54E-09 | D7 |
| ko05135:Infectious disease: bacterial | 1.18 | 1.57E-09 | D7 |
| ko05204:Cancer: overview | 1.17 | 4.28E-07 | D7 |
| ko05225:Cancer: specific types | 1.08 | 1.85E-05 | D7 |
| ko04622:Immune system | 0.91 | 2.98E-03 | D7 |
| ko00364:Xenobiotics biodegradation and metabolism | 0.89 | 1.21E-04 | D7 |
| ko05219:Cancer: specific types | 0.62 | 4.51E-07 | D7 |
| ko00100:Lipid metabolism | 0.54 | 8.05E-07 | D7 |
| ko00909:Metabolism of terpenoids and polyketides | 0.53 | 5.56E-06 | D7 |
| ko00624:Xenobiotics biodegradation and metabolism | 0.35 | 3.21E-10 | D7 |

**Supplementary Table 6.** Different enriched KOs and KEGG pathways among different time points of LAN stud boar samples.

| **Item** | **LDA Score** | ***p*-value (corrected)** | **Group** |
| --- | --- | --- | --- |
| ko02040:Cell motility | 3.17 | 4.75E-09 | D0 |
| ko02020:Signal transduction | 3.14 | 9.94E-11 | D0 |
| ko02030:Cell motility | 3.09 | 5.03E-10 | D0 |
| ko00860:Metabolism of cofactors and vitamins | 2.98 | 1.91E-08 | D0 |
| ko00190:Energy metabolism | 2.54 | 1.13E-09 | D0 |
| ko01212:Global and overview maps | 2.38 | 1.31E-09 | D0 |
| ko00720:Energy metabolism | 2.37 | 3.87E-03 | D0 |
| ko04626:Environmental adaptation | 2.33 | 2.14E-08 | D0 |
| ko04621:Immune system | 2.3 | 9.57E-11 | D0 |
| ko04122:Folding, sorting and degradation | 2.28 | 2.59E-10 | D0 |
| ko05132:Infectious disease: bacterial | 2.25 | 1.84E-08 | D0 |
| ko00650:Carbohydrate metabolism | 2.21 | 4.19E-03 | D0 |
| ko00340:Amino acid metabolism | 2.2 | 2.09E-05 | D0 |
| ko03018:Folding, sorting and degradation | 2.17 | 7.65E-06 | D0 |
| ko00410:Metabolism of other amino acids | 2.13 | 3.24E-10 | D0 |
| ko04152:Signal transduction | 2.12 | 2.55E-07 | D0 |
| ko00780:Metabolism of cofactors and vitamins | 2.12 | 1.32E-04 | D0 |
| ko00333:Biosynthesis of other secondary metabolites | 2.02 | 1.25E-06 | D0 |
| ko04141:Folding, sorting and degradation | 2.01 | 2.10E-05 | D0 |
| ko00310:Amino acid metabolism | 1.96 | 3.43E-05 | D0 |
| ko00061:Lipid metabolism | 1.92 | 5.51E-05 | D0 |
| ko01051:Metabolism of terpenoids and polyketides | 1.91 | 2.28E-04 | D0 |
| ko04727:Nervous system | 1.91 | 2.20E-11 | D0 |
| ko04151:Signal transduction | 1.9 | 3.18E-05 | D0 |
| ko05418:Cardiovascular disease | 1.88 | 2.14E-05 | D0 |
| ko00770:Metabolism of cofactors and vitamins | 1.86 | 4.52E-04 | D0 |
| ko00380:Amino acid metabolism | 1.82 | 1.27E-05 | D0 |
| ko00460:Metabolism of other amino acids | 1.81 | 3.15E-02 | D0 |
| ko05206:Cancer: overview | 1.8 | 1.33E-09 | D0 |
| ko05134:Infectious disease: bacterial | 1.8 | 4.89E-05 | D0 |
| ko05131:Infectious disease: bacterial | 1.79 | 1.01E-04 | D0 |
| ko00072:Lipid metabolism | 1.77 | 4.42E-07 | D0 |
| ko05215:Cancer: specific types | 1.77 | 1.71E-05 | D0 |
| ko04914:Endocrine system | 1.77 | 1.71E-05 | D0 |
| ko04612:Immune system | 1.77 | 1.71E-05 | D0 |
| ko04657:Immune system | 1.77 | 1.71E-05 | D0 |
| ko04659:Immune system | 1.77 | 1.71E-05 | D0 |
| ko04915:Endocrine system | 1.77 | 1.71E-05 | D0 |
| ko00523:Metabolism of terpenoids and polyketides | 1.77 | 8.96E-03 | D0 |
| ko01502:Drug resistance: antimicrobial | 1.77 | 6.08E-03 | D0 |
| ko00524:Biosynthesis of other secondary metabolites | 1.77 | 9.63E-11 | D0 |
| ko00998:Biosynthesis of other secondary metabolites | 1.76 | 2.52E-05 | D0 |
| ko04964:Excretory system | 1.72 | 1.85E-10 | D0 |
| ko02025:Cellular community - prokaryotes | 1.68 | 1.28E-02 | D0 |
| ko00280:Amino acid metabolism | 1.66 | 1.54E-02 | D0 |
| ko05200:Cancer: overview | 1.62 | 2.33E-02 | D0 |
| ko00950:Biosynthesis of other secondary metabolites | 1.53 | 3.89E-03 | D0 |
| ko00401:Biosynthesis of other secondary metabolites | 1.47 | 6.41E-03 | D0 |
| ko04361:Development and regeneration | 1.46 | 2.40E-04 | D0 |
| ko04072:Signal transduction | 1.46 | 2.40E-04 | D0 |
| ko00525:Biosynthesis of other secondary metabolites | 1.45 | 2.44E-02 | D0 |
| ko05231:Cancer: overview | 1.44 | 3.10E-04 | D0 |
| ko04068:Signal transduction | 1.44 | 7.38E-03 | D0 |
| ko00966:Biosynthesis of other secondary metabolites | 1.43 | 2.77E-04 | D0 |
| ko00960:Biosynthesis of other secondary metabolites | 1.35 | 4.19E-02 | D0 |
| ko00643:Xenobiotics biodegradation and metabolism | 1.23 | 3.46E-02 | D0 |
| ko04214:Cell growth and death | 1.05 | 4.73E-02 | D0 |
| ko03450:Replication and repair | 0.99 | 3.76E-04 | D0 |
| ko05145:Infectious disease: parasitic | 0.94 | 2.14E-11 | D0 |
| ko03022:Transcription | 0.88 | 1.89E-03 | D0 |
| ko05012:Neurodegenerative disease | 0.83 | 1.14E-09 | D0 |
| ko04932:Endocrine and metabolic disease | 0.83 | 1.22E-09 | D0 |
| ko04113:Cell growth and death | 0.82 | 1.82E-02 | D0 |
| ko00906:Metabolism of terpenoids and polyketides | 0.77 | 5.54E-03 | D0 |
| ko05416:Cardiovascular disease | 0.62 | 2.75E-08 | D0 |
| ko05163:Infectious disease: viral | 0.62 | 4.08E-08 | D0 |
| ko05170:Infectious disease: viral | 0.62 | 4.08E-08 | D0 |
| ko05167:Infectious disease: viral | 0.62 | 4.08E-08 | D0 |
| ko04215:Cell growth and death | 0.62 | 4.08E-08 | D0 |
| ko04115:Cell growth and death | 0.62 | 4.08E-08 | D0 |
| ko05210:Cancer: specific types | 0.62 | 4.08E-08 | D0 |
| ko05222:Cancer: specific types | 0.62 | 4.08E-08 | D0 |
| ko05169:Infectious disease: viral | 0.62 | 4.08E-08 | D0 |
| ko05161:Infectious disease: viral | 0.62 | 4.08E-08 | D0 |
| ko05160:Infectious disease: viral | 0.62 | 4.08E-08 | D0 |
| ko05168:Infectious disease: viral | 0.62 | 4.08E-08 | D0 |
| ko05162:Infectious disease: viral | 0.62 | 4.08E-08 | D0 |
| ko05164:Infectious disease: viral | 0.62 | 4.08E-08 | D0 |
| ko00140:Lipid metabolism | 0.61 | 1.50E-04 | D0 |
| ko04614:Endocrine system | 0.59 | 4.56E-03 | D0 |
| ko04260:Circulatory system | 0.43 | 1.15E-07 | D0 |
| ko00941:Biosynthesis of other secondary metabolites | 0.3 | 1.73E-02 | D0 |
| ko00945:Biosynthesis of other secondary metabolites | 0.3 | 1.73E-02 | D0 |
| ko04723:Nervous system | 0.29 | 4.05E-13 | D0 |
| ko05017:Neurodegenerative disease | 0.19 | 2.63E-02 | D0 |
| ko05110:Infectious disease: bacterial | 0.16 | 1.67E-06 | D0 |
| ko04640:Immune system | 0.07 | 4.58E-02 | D0 |
| ko00943:Biosynthesis of other secondary metabolites | 0.06 | 3.74E-04 | D0 |
| ko00232:Biosynthesis of other secondary metabolites | 0.04 | 1.04E-02 | D0 |
| ko04111:Cell growth and death | 0.03 | 8.96E-03 | D0 |
| ko01057:Metabolism of terpenoids and polyketides | 0.03 | 9.10E-03 | D0 |
| ko00365:Xenobiotics biodegradation and metabolism | 0.02 | 3.93E-04 | D0 |
| ko00620:Carbohydrate metabolism | 2.61 | 8.70E-11 | D14 |
| ko00240:Nucleotide metabolism | 2.53 | 2.59E-10 | D14 |
| ko00010:Carbohydrate metabolism | 2.5 | 1.14E-04 | D14 |
| ko03430:Replication and repair | 2.43 | 1.19E-13 | D14 |
| ko00230:Nucleotide metabolism | 2.4 | 3.93E-04 | D14 |
| ko00730:Metabolism of cofactors and vitamins | 2.36 | 1.04E-11 | D14 |
| ko01200:Global and overview maps | 2.29 | 4.78E-03 | D14 |
| ko05111:Cellular community - prokaryotes | 2.23 | 5.97E-05 | D14 |
| ko00480:Metabolism of other amino acids | 2.21 | 1.43E-03 | D14 |
| ko00900:Metabolism of terpenoids and polyketides | 2.18 | 1.01E-09 | D14 |
| ko05230:Cancer: overview | 2.18 | 4.36E-04 | D14 |
| ko00983:Xenobiotics biodegradation and metabolism | 2.16 | 1.01E-10 | D14 |
| ko00250:Amino acid metabolism | 2.16 | 3.75E-05 | D14 |
| ko00564:Lipid metabolism | 2.15 | 1.55E-07 | D14 |
| ko04066:Signal transduction | 2.14 | 9.62E-07 | D14 |
| ko04112:Cell growth and death | 2.12 | 8.44E-10 | D14 |
| ko04922:Endocrine system | 2.12 | 2.73E-04 | D14 |
| ko00640:Carbohydrate metabolism | 2.12 | 4.61E-04 | D14 |
| ko00910:Energy metabolism | 2.11 | 1.68E-04 | D14 |
| ko00561:Lipid metabolism | 2.08 | 2.34E-03 | D14 |
| ko00330:Amino acid metabolism | 2.06 | 2.02E-03 | D14 |
| ko00680:Energy metabolism | 2.05 | 1.26E-04 | D14 |
| ko03420:Replication and repair | 2.05 | 4.08E-08 | D14 |
| ko05150:Infectious disease: bacterial | 1.99 | 4.58E-04 | D14 |
| ko03013:Translation | 1.98 | 6.38E-14 | D14 |
| ko00633:Xenobiotics biodegradation and metabolism | 1.92 | 2.04E-09 | D14 |
| ko04724:Nervous system | 1.87 | 9.17E-11 | D14 |
| ko00121:Lipid metabolism | 1.83 | 2.02E-07 | D14 |
| ko01503:Drug resistance: antimicrobial | 1.81 | 1.50E-02 | D14 |
| ko00473:Metabolism of other amino acids | 1.81 | 5.94E-04 | D14 |
| ko03060:Folding, sorting and degradation | 1.78 | 1.55E-02 | D14 |
| ko00626:Xenobiotics biodegradation and metabolism | 1.73 | 1.90E-03 | D14 |
| ko00625:Xenobiotics biodegradation and metabolism | 1.7 | 3.09E-03 | D14 |
| ko00350:Amino acid metabolism | 1.7 | 2.83E-03 | D14 |
| ko05203:Cancer: overview | 1.69 | 1.50E-12 | D14 |
| ko05165:Infectious disease: viral | 1.69 | 1.50E-12 | D14 |
| ko03410:Replication and repair | 1.68 | 4.56E-03 | D14 |
| ko04930:Endocrine and metabolic disease | 1.67 | 3.55E-10 | D14 |
| ko00120:Lipid metabolism | 1.57 | 2.58E-04 | D14 |
| ko00471:Metabolism of other amino acids | 1.56 | 7.52E-06 | D14 |
| ko01054:Metabolism of terpenoids and polyketides | 1.54 | 5.27E-07 | D14 |
| ko00980:Xenobiotics biodegradation and metabolism | 1.52 | 3.49E-03 | D14 |
| ko00982:Xenobiotics biodegradation and metabolism | 1.52 | 3.57E-03 | D14 |
| ko00830:Metabolism of cofactors and vitamins | 1.52 | 3.06E-03 | D14 |
| ko00622:Xenobiotics biodegradation and metabolism | 1.51 | 2.65E-03 | D14 |
| ko00621:Xenobiotics biodegradation and metabolism | 1.44 | 2.48E-03 | D14 |
| ko00627:Xenobiotics biodegradation and metabolism | 1.4 | 2.33E-02 | D14 |
| ko00430:Metabolism of other amino acids | 1.39 | 3.30E-04 | D14 |
| ko04978:Digestive system | 1.32 | 1.52E-08 | D14 |
| ko00791:Xenobiotics biodegradation and metabolism | 1.23 | 1.40E-06 | D14 |
| ko00311:Biosynthesis of other secondary metabolites | 1.23 | 2.65E-03 | D14 |
| ko05211:Cancer: specific types | 1.22 | 2.04E-03 | D14 |
| ko04934:Endocrine and metabolic disease | 1.22 | 2.04E-03 | D14 |
| ko00472:Metabolism of other amino acids | 1.17 | 2.48E-03 | D14 |
| ko04622:Immune system | 0.9 | 1.02E-04 | D14 |
| ko04919:Endocrine system | 0.72 | 2.38E-17 | D14 |
| ko00565:Lipid metabolism | 0.6 | 1.05E-03 | D14 |
| ko04011:Signal transduction | 0.51 | 1.71E-03 | D14 |
| ko00984:Xenobiotics biodegradation and metabolism | 0.49 | 1.07E-16 | D14 |
| ko04024:Signal transduction | 0.26 | 3.99E-15 | D14 |
| ko04071:Signal transduction | 0.23 | 1.07E-16 | D14 |
| ko05034:Substance dependence | 0.19 | 1.54E-06 | D14 |
| ko05031:Substance dependence | 0.19 | 1.54E-06 | D14 |
| ko04728:Nervous system | 0.19 | 1.54E-06 | D14 |
| ko05030:Substance dependence | 0.19 | 1.62E-06 | D14 |
| ko04726:Nervous system | 0.19 | 1.62E-06 | D14 |
| ko00997:Biosynthesis of other secondary metabolites | 0.16 | 2.09E-10 | D14 |
| ko04976:Digestive system | 0.04 | 1.27E-05 | D14 |
| ko04928:Endocrine system | 0.03 | 6.21E-04 | D14 |
| ko01100:Global and overview maps | 3.19 | 1.38E-09 | D7 |
| ko01110:Global and overview maps | 3.01 | 9.77E-10 | D7 |
| ko01230:Global and overview maps | 3.01 | 8.18E-06 | D7 |
| ko02010:Membrane transport | 2.95 | 1.69E-03 | D7 |
| ko02060:Membrane transport | 2.81 | 5.47E-03 | D7 |
| ko00540:Glycan biosynthesis and metabolism | 2.64 | 4.52E-04 | D7 |
| ko00520:Carbohydrate metabolism | 2.58 | 2.50E-04 | D7 |
| ko00051:Carbohydrate metabolism | 2.56 | 1.07E-04 | D7 |
| ko01210:Global and overview maps | 2.55 | 1.19E-07 | D7 |
| ko02024:Cellular community - prokaryotes | 2.5 | 3.58E-02 | D7 |
| ko00260:Amino acid metabolism | 2.48 | 5.18E-12 | D7 |
| ko00052:Carbohydrate metabolism | 2.48 | 3.14E-03 | D7 |
| ko00400:Amino acid metabolism | 2.47 | 2.09E-07 | D7 |
| ko01501:Drug resistance: antimicrobial | 2.47 | 2.52E-06 | D7 |
| ko00760:Metabolism of cofactors and vitamins | 2.4 | 6.43E-11 | D7 |
| ko00270:Amino acid metabolism | 2.39 | 2.13E-07 | D7 |
| ko00290:Amino acid metabolism | 2.37 | 4.08E-08 | D7 |
| ko00660:Carbohydrate metabolism | 2.32 | 2.96E-12 | D7 |
| ko00020:Carbohydrate metabolism | 2.29 | 1.11E-03 | D7 |
| ko01220:Global and overview maps | 2.22 | 2.24E-05 | D7 |
| ko00130:Metabolism of cofactors and vitamins | 2.21 | 3.09E-03 | D7 |
| ko00053:Carbohydrate metabolism | 2.21 | 9.59E-10 | D7 |
| ko04146:Transport and catabolism | 2.18 | 1.42E-06 | D7 |
| ko00750:Metabolism of cofactors and vitamins | 2.15 | 7.92E-13 | D7 |
| ko00040:Carbohydrate metabolism | 2.13 | 7.23E-06 | D7 |
| ko04972:Digestive system | 2.1 | 1.85E-10 | D7 |
| ko04973:Digestive system | 2.1 | 7.90E-12 | D7 |
| ko00630:Carbohydrate metabolism | 2.09 | 4.33E-02 | D7 |
| ko00300:Amino acid metabolism | 2.07 | 3.63E-03 | D7 |
| ko04714:Environmental adaptation | 2.05 | 7.31E-06 | D7 |
| ko00710:Energy metabolism | 2.03 | 2.74E-05 | D7 |
| ko00261:Biosynthesis of other secondary metabolites | 2 | 7.65E-06 | D7 |
| ko04920:Endocrine system | 1.99 | 4.09E-04 | D7 |
| ko00970:Translation | 1.97 | 4.57E-02 | D7 |
| ko00450:Metabolism of other amino acids | 1.92 | 6.67E-05 | D7 |
| ko00940:Biosynthesis of other secondary metabolites | 1.92 | 3.18E-05 | D7 |
| ko00572:Glycan biosynthesis and metabolism | 1.9 | 8.86E-10 | D7 |
| ko00603:Glycan biosynthesis and metabolism | 1.88 | 1.30E-05 | D7 |
| ko04216:Cell growth and death | 1.88 | 1.76E-02 | D7 |
| ko00405:Biosynthesis of other secondary metabolites | 1.86 | 2.13E-13 | D7 |
| ko00571:Glycan biosynthesis and metabolism | 1.8 | 7.44E-19 | D7 |
| ko00562:Carbohydrate metabolism | 1.79 | 8.48E-04 | D7 |
| ko04917:Endocrine system | 1.77 | 2.52E-05 | D7 |
| ko01053:Metabolism of terpenoids and polyketides | 1.73 | 1.13E-09 | D7 |
| ko00220:Amino acid metabolism | 1.72 | 2.63E-02 | D7 |
| ko05340:Immune disease | 1.7 | 2.28E-04 | D7 |
| ko05100:Infectious disease: bacterial | 1.67 | 3.77E-10 | D7 |
| ko05143:Infectious disease: parasitic | 1.66 | 9.74E-09 | D7 |
| ko00361:Xenobiotics biodegradation and metabolism | 1.65 | 1.62E-09 | D7 |
| ko03020:Transcription | 1.64 | 2.42E-03 | D7 |
| ko01040:Lipid metabolism | 1.63 | 6.38E-14 | D7 |
| ko00785:Metabolism of cofactors and vitamins | 1.63 | 1.00E-04 | D7 |
| ko00531:Glycan biosynthesis and metabolism | 1.63 | 7.38E-04 | D7 |
| ko05133:Infectious disease: bacterial | 1.61 | 7.70E-05 | D7 |
| ko00332:Biosynthesis of other secondary metabolites | 1.59 | 3.68E-11 | D7 |
| ko03008:Translation | 1.57 | 1.27E-08 | D7 |
| ko00510:Glycan biosynthesis and metabolism | 1.57 | 2.14E-08 | D7 |
| ko04217:Cell growth and death | 1.52 | 2.21E-02 | D7 |
| ko00440:Metabolism of other amino acids | 1.51 | 1.12E-04 | D7 |
| ko03050:Folding, sorting and degradation | 1.5 | 2.24E-19 | D7 |
| ko05152:Infectious disease: bacterial | 1.48 | 2.49E-04 | D7 |
| ko05142:Infectious disease: parasitic | 1.48 | 2.83E-08 | D7 |
| ko04070:Signal transduction | 1.46 | 1.39E-03 | D7 |
| ko01524:Drug resistance: antineoplastic | 1.4 | 2.46E-02 | D7 |
| ko04931:Endocrine and metabolic disease | 1.38 | 5.30E-04 | D7 |
| ko04974:Digestive system | 1.37 | 1.81E-02 | D7 |
| ko04910:Endocrine system | 1.37 | 7.44E-03 | D7 |
| ko05135:Infectious disease: bacterial | 1.32 | 4.21E-09 | D7 |
| ko05204:Cancer: overview | 1.29 | 2.47E-09 | D7 |
| ko05225:Cancer: specific types | 1.15 | 5.05E-07 | D7 |
| ko00965:Biosynthesis of other secondary metabolites | 1.12 | 2.32E-04 | D7 |
| ko05020:Neurodegenerative disease | 1.12 | 1.53E-06 | D7 |
| ko00944:Biosynthesis of other secondary metabolites | 1.1 | 3.75E-05 | D7 |
| ko05130:Infectious disease: bacterial | 1.08 | 1.48E-03 | D7 |
| ko00281:Metabolism of terpenoids and polyketides | 1.08 | 8.96E-03 | D7 |
| ko00930:Xenobiotics biodegradation and metabolism | 0.94 | 4.62E-06 | D7 |
| ko00364:Xenobiotics biodegradation and metabolism | 0.94 | 1.36E-08 | D7 |
| ko00592:Lipid metabolism | 0.93 | 6.09E-04 | D7 |
| ko00642:Xenobiotics biodegradation and metabolism | 0.84 | 7.70E-05 | D7 |
| ko05219:Cancer: specific types | 0.78 | 5.93E-11 | D7 |
| ko00623:Xenobiotics biodegradation and metabolism | 0.75 | 1.28E-02 | D7 |
| ko00100:Lipid metabolism | 0.47 | 1.54E-06 | D7 |
| ko00909:Metabolism of terpenoids and polyketides | 0.47 | 2.27E-06 | D7 |
| ko00624:Xenobiotics biodegradation and metabolism | 0.24 | 3.28E-08 | D7 |
| ko04144:Transport and catabolism | 0.03 | 8.53E-04 | D7 |

**Supplementary Table 7.** Different enriched KOs and KEGG pathways among different time points of DUR stud boar samples.

| **Item** | **LDA Score** | ***p*-value (corrected)** | **Group** |
| --- | --- | --- | --- |
| ko01100:Global and overview maps | 3.23 | 2.68E-03 | D0 |
| ko01110:Global and overview maps | 3.19 | 2.29E-03 | D0 |
| ko01230:Global and overview maps | 3.19 | 1.84E-02 | D0 |
| ko00860:Metabolism of cofactors and vitamins | 2.99 | 6.13E-03 | D0 |
| ko00400:Amino acid metabolism | 2.84 | 5.59E-03 | D0 |
| ko01210:Global and overview maps | 2.77 | 1.01E-02 | D0 |
| ko00540:Glycan biosynthesis and metabolism | 2.7 | 3.16E-02 | D0 |
| ko00340:Amino acid metabolism | 2.66 | 7.90E-03 | D0 |
| ko00190:Energy metabolism | 2.58 | 4.19E-03 | D0 |
| ko00290:Amino acid metabolism | 2.55 | 1.78E-02 | D0 |
| ko00720:Energy metabolism | 2.49 | 1.48E-02 | D0 |
| ko00195:Energy metabolism | 2.43 | 1.81E-03 | D0 |
| ko00910:Energy metabolism | 2.4 | 2.42E-03 | D0 |
| ko00220:Amino acid metabolism | 2.38 | 2.06E-02 | D0 |
| ko00660:Carbohydrate metabolism | 2.37 | 7.88E-03 | D0 |
| ko00770:Metabolism of cofactors and vitamins | 2.34 | 6.13E-03 | D0 |
| ko00330:Amino acid metabolism | 2.27 | 2.68E-03 | D0 |
| ko00730:Metabolism of cofactors and vitamins | 2.26 | 3.48E-02 | D0 |
| ko04217:Cell growth and death | 2.23 | 1.81E-03 | D0 |
| ko00940:Biosynthesis of other secondary metabolites | 2.2 | 1.39E-02 | D0 |
| ko00740:Metabolism of cofactors and vitamins | 2.15 | 3.55E-02 | D0 |
| ko00460:Metabolism of other amino acids | 2.13 | 1.75E-02 | D0 |
| ko00401:Biosynthesis of other secondary metabolites | 2.12 | 3.92E-03 | D0 |
| ko00360:Amino acid metabolism | 2.11 | 4.80E-02 | D0 |
| ko05120:Infectious disease: bacterial | 2.1 | 2.68E-03 | D0 |
| ko00960:Biosynthesis of other secondary metabolites | 2.09 | 5.93E-03 | D0 |
| ko00750:Metabolism of cofactors and vitamins | 2.03 | 1.78E-02 | D0 |
| ko00333:Biosynthesis of other secondary metabolites | 1.99 | 4.58E-03 | D0 |
| ko04931:Endocrine and metabolic disease | 1.91 | 5.17E-03 | D0 |
| ko00966:Biosynthesis of other secondary metabolites | 1.89 | 1.78E-02 | D0 |
| ko00950:Biosynthesis of other secondary metabolites | 1.88 | 1.42E-02 | D0 |
| ko00405:Biosynthesis of other secondary metabolites | 1.78 | 1.19E-02 | D0 |
| ko04724:Nervous system | 1.73 | 1.74E-02 | D0 |
| ko04151:Signal transduction | 1.72 | 2.97E-02 | D0 |
| ko04210:Cell growth and death | 1.71 | 3.07E-02 | D0 |
| ko00633:Xenobiotics biodegradation and metabolism | 1.69 | 3.33E-02 | D0 |
| ko00998:Biosynthesis of other secondary metabolites | 1.65 | 2.97E-02 | D0 |
| ko00590:Lipid metabolism | 1.63 | 1.19E-02 | D0 |
| ko05215:Cancer: specific types | 1.63 | 6.74E-03 | D0 |
| ko04915:Endocrine system | 1.63 | 6.74E-03 | D0 |
| ko04914:Endocrine system | 1.63 | 6.74E-03 | D0 |
| ko04612:Immune system | 1.63 | 6.74E-03 | D0 |
| ko04657:Immune system | 1.63 | 6.74E-03 | D0 |
| ko04659:Immune system | 1.63 | 6.74E-03 | D0 |
| ko04978:Digestive system | 1.47 | 3.91E-03 | D0 |
| ko05322:Immune disease | 0.36 | 8.85E-03 | D0 |
| ko00500:Carbohydrate metabolism | 3.02 | 4.14E-02 | D14 |
| ko00010:Carbohydrate metabolism | 2.91 | 2.68E-03 | D14 |
| ko03010:Translation | 2.84 | 2.28E-02 | D14 |
| ko00230:Nucleotide metabolism | 2.73 | 2.25E-02 | D14 |
| ko00561:Lipid metabolism | 2.67 | 5.27E-03 | D14 |
| ko00030:Carbohydrate metabolism | 2.63 | 1.81E-03 | D14 |
| ko05230:Cancer: overview | 2.61 | 5.59E-03 | D14 |
| ko00620:Carbohydrate metabolism | 2.57 | 1.78E-02 | D14 |
| ko00970:Translation | 2.54 | 3.76E-03 | D14 |
| ko00300:Amino acid metabolism | 2.49 | 2.61E-02 | D14 |
| ko04066:Signal transduction | 2.44 | 7.04E-03 | D14 |
| ko04922:Endocrine system | 2.43 | 2.73E-02 | D14 |
| ko05150:Infectious disease: bacterial | 2.37 | 3.02E-03 | D14 |
| ko03030:Replication and repair | 2.34 | 1.39E-02 | D14 |
| ko03440:Replication and repair | 2.32 | 4.17E-02 | D14 |
| ko03430:Replication and repair | 2.29 | 1.66E-02 | D14 |
| ko03420:Replication and repair | 2.13 | 1.01E-02 | D14 |
| ko03410:Replication and repair | 2.12 | 3.92E-03 | D14 |
| ko03018:Folding, sorting and degradation | 2.1 | 4.17E-02 | D14 |
| ko03060:Folding, sorting and degradation | 2.09 | 3.16E-02 | D14 |
| ko03020:Transcription | 2.07 | 2.68E-03 | D14 |
| ko00473:Metabolism of other amino acids | 1.98 | 2.35E-02 | D14 |
| ko00072:Lipid metabolism | 1.95 | 1.75E-02 | D14 |
| ko00120:Lipid metabolism | 1.94 | 1.86E-02 | D14 |
| ko05340:Immune disease | 1.87 | 1.72E-03 | D14 |
| ko04016:Signal transduction | 1.83 | 1.19E-02 | D14 |
| ko00830:Metabolism of cofactors and vitamins | 1.77 | 3.29E-02 | D14 |
| ko00622:Xenobiotics biodegradation and metabolism | 1.76 | 8.18E-03 | D14 |
| ko03013:Translation | 1.76 | 3.03E-02 | D14 |
| ko01524:Drug resistance: antineoplastic | 1.74 | 5.73E-03 | D14 |
| ko05211:Cancer: specific types | 1.7 | 3.92E-03 | D14 |
| ko04934:Endocrine and metabolic disease | 1.7 | 3.92E-03 | D14 |
| ko00621:Xenobiotics biodegradation and metabolism | 1.69 | 9.58E-03 | D14 |
| ko04940:Endocrine and metabolic disease | 1.56 | 6.74E-03 | D14 |
| ko05205:Cancer: overview | 1.18 | 1.09E-02 | D14 |
| ko05145:Infectious disease: parasitic | 1.14 | 3.92E-03 | D14 |
| ko00571:Glycan biosynthesis and metabolism | 0.93 | 2.42E-03 | D14 |
| ko03450:Replication and repair | 0.88 | 3.16E-02 | D14 |
| ko04976:Digestive system | 0.42 | 1.78E-02 | D14 |
| ko00984:Xenobiotics biodegradation and metabolism | 0.35 | 6.44E-04 | D14 |
| ko03050:Folding, sorting and degradation | 0.35 | 3.92E-03 | D14 |
| ko04071:Signal transduction | 0.31 | 1.65E-04 | D14 |
| ko04024:Signal transduction | 0.25 | 6.44E-04 | D14 |
| ko02060:Membrane transport | 3.26 | 6.74E-03 | D7 |
| ko02020:Signal transduction | 2.94 | 6.55E-03 | D7 |
| ko01503:Drug resistance: antimicrobial | 2.4 | 1.49E-02 | D7 |
| ko00480:Metabolism of other amino acids | 2.39 | 4.20E-02 | D7 |
| ko00130:Metabolism of cofactors and vitamins | 2.32 | 3.85E-02 | D7 |
| ko05111:Cellular community - prokaryotes | 2.3 | 6.74E-03 | D7 |
| ko02026:Cellular community - prokaryotes | 2.24 | 1.19E-02 | D7 |
| ko00920:Energy metabolism | 2.17 | 4.68E-02 | D7 |
| ko00053:Carbohydrate metabolism | 2.08 | 6.16E-03 | D7 |
| ko05133:Infectious disease: bacterial | 2.05 | 5.21E-03 | D7 |
| ko00627:Xenobiotics biodegradation and metabolism | 2.03 | 1.75E-02 | D7 |
| ko05131:Infectious disease: bacterial | 2.01 | 1.19E-02 | D7 |
| ko00430:Metabolism of other amino acids | 1.97 | 1.75E-02 | D7 |
| ko00380:Amino acid metabolism | 1.95 | 2.35E-02 | D7 |
| ko05100:Infectious disease: bacterial | 1.94 | 2.16E-03 | D7 |
| ko00440:Metabolism of other amino acids | 1.93 | 2.42E-03 | D7 |
| ko05016:Neurodegenerative disease | 1.92 | 1.46E-02 | D7 |
| ko00980:Xenobiotics biodegradation and metabolism | 1.92 | 6.69E-03 | D7 |
| ko00982:Xenobiotics biodegradation and metabolism | 1.91 | 6.74E-03 | D7 |
| ko01053:Metabolism of terpenoids and polyketides | 1.81 | 6.55E-03 | D7 |
| ko05014:Neurodegenerative disease | 1.8 | 8.57E-03 | D7 |
| ko00785:Metabolism of cofactors and vitamins | 1.75 | 2.76E-02 | D7 |
| ko04070:Signal transduction | 1.73 | 2.42E-03 | D7 |
| ko04972:Digestive system | 1.7 | 7.51E-04 | D7 |
| ko04973:Digestive system | 1.68 | 2.29E-03 | D7 |
| ko00281:Metabolism of terpenoids and polyketides | 1.61 | 1.81E-03 | D7 |
| ko05135:Infectious disease: bacterial | 1.59 | 1.81E-03 | D7 |
| ko05204:Cancer: overview | 1.56 | 1.81E-03 | D7 |
| ko05225:Cancer: specific types | 1.54 | 2.16E-03 | D7 |
| ko05020:Neurodegenerative disease | 1.45 | 2.15E-03 | D7 |
| ko01040:Lipid metabolism | 1.43 | 2.16E-03 | D7 |
| ko00592:Lipid metabolism | 1.4 | 2.29E-03 | D7 |
| ko00623:Xenobiotics biodegradation and metabolism | 1.36 | 5.21E-03 | D7 |
| ko00930:Xenobiotics biodegradation and metabolism | 1.34 | 2.16E-03 | D7 |
| ko00364:Xenobiotics biodegradation and metabolism | 1.29 | 2.42E-03 | D7 |
| ko00642:Xenobiotics biodegradation and metabolism | 1.22 | 1.81E-03 | D7 |
| ko00591:Lipid metabolism | 1.05 | 5.59E-03 | D7 |
| ko00140:Lipid metabolism | 1.04 | 1.02E-02 | D7 |
| ko00944:Biosynthesis of other secondary metabolites | 1.03 | 2.88E-03 | D7 |
| ko05143:Infectious disease: parasitic | 1.03 | 1.72E-02 | D7 |
| ko05142:Infectious disease: parasitic | 1.01 | 1.13E-02 | D7 |
| ko00965:Biosynthesis of other secondary metabolites | 1.01 | 3.51E-03 | D7 |
| ko00565:Lipid metabolism | 1 | 6.74E-03 | D7 |
| ko05219:Cancer: specific types | 0.92 | 2.68E-03 | D7 |
| ko04113:Cell growth and death | 0.75 | 8.71E-04 | D7 |
| ko00906:Metabolism of terpenoids and polyketides | 0.73 | 3.92E-03 | D7 |
| ko03022:Transcription | 0.72 | 3.92E-03 | D7 |
| ko04011:Signal transduction | 0.64 | 3.16E-02 | D7 |
| ko00909:Metabolism of terpenoids and polyketides | 0.42 | 6.44E-04 | D7 |
| ko00100:Lipid metabolism | 0.42 | 6.44E-04 | D7 |
